# Liquid Crystals as Stationary Phases in Chromatography

**DOI:** 10.1007/s10337-016-3154-5

**Published:** 2016-08-13

**Authors:** H. Grajek, Z. Witkiewicz, M. Purchała, W. Drzewiński

**Affiliations:** Institute of Chemistry, Military University of Technology, Warsaw, Poland

**Keywords:** Liquid crystals, Liquid crystalline stationary phases, Acceptor–donor properties of liquid crystalline stationary phases, Inverse gas chromatography

## Abstract

The most correct analysis of the compositions of diverse analytes mixtures is significant for analytical studies in different fields; however, many prevalent analytes cannot be identified employing traditional partition gas chromatographic methods. Thus, the increasing requirements on analytes of isomeric compounds and the problems encountered in their separation demand a study of more diverse analytical systems which are characterised by higher selectivity. Therefore, the selectivity and polarities of various liquid crystals (rod-like, banana-shape, biforked, oxygen, sulphur, nitrogen, and metal containing molecules, Schiff-base, and polymeric dendrimers) employed as liquid crystalline stationary phases (LCSPs) have been discussed from both points of views, namely, their analytical applications and thermodynamic characteristics of infinitely diluted probes with different acceptor–donor properties. Extreme particular effort has been paid to the different interdependencies between the bound up chemical structures of liquid crystal molecules with their different acceptor–donor properties and the connected resolution capabilities in the interpretation of the probe—LCSP systems, on the basis of the ${ \ln }V_{g\left( T \right)} = f\left( {\frac{1}{T}} \right)$ and ${ \ln }\left( {\frac{{a_{1} }}{{w_{1} }}} \right)^{\infty } = f\left( {\frac{1}{T}} \right)$ dependencies, with regard to the LCSP compositions, which have been controlled by the counterbalancing of the enthalpy and entropy factors. The properties of binary systems composed of liquid crystalline poly(propyleneimine) dendrimers—rod-like molecules of liquid crystals and effects of the dendrimer structure, the chemical nature, and molecular size of the non-mesogens on the ability to dissolve in the liquid crystalline phases, have been interpreted. Practical applications of metallomesogenes and chiral stationary phases for analytical separation of different organic substances have also been taken into consideration.

## Introduction

The application of liquid crystals as stationary phases in gas chromatography was reported for the first time by Kelker [1–3], and Dewar and Schroeder [4, 5]. They were initially employed in gas chromatography and then also in liquid and supercritical chromatography. They have also been used for the visualisation of thin-layer chromatograms. The liquid crystals thermodynamic properties have been tested by means of different chromatographic techniques, for instance, their phase transitions and the thermodynamic effects of a solution of chromatographed substances in the liquid crystals mesophases. These problems have been scrutinised in some articles encompassing the state-of-the-art methods until the year 2000 [6–10]. This interesting review is devoted to the investigation of liquid crystalline metal complexes as column packings with different acceptor–donor properties in gas chromatography [11]. According to the authors, electron-donor interactions were influenced by factors originating from the probe molecules (i.e., linear and branched aliphatic hydrocarbons, aromatic and cyclic hydrocarbons, halogenated hydrocarbons, ethers, and thioethers) and from the column fillings.

While testing the impact of the configuration of the probe molecules on the specific interactions, the following factors have been taken into consideration: the quantity of unsaturated bonds and their types and positions in a molecule, the number and types of substituents and the presence of heteroatoms (S, O) in the molecules [11].

In this review, based on the existing background of the overall knowledge concerning the liquid crystals properties as chromatographic stationary phases described in papers [2–7], we interpret the chromatographic results of the liquid crystals testing undertaken over a period of approximately the last 10 years. Moreover, we also attempt to anticipate the courses of investigations of the liquid crystals stationary phases (LCSPs).

The cyclization kinetics of a new phenyldiazene liquid crystal with an activated methylene group in the position *ortho* to the diazo linkage was studied by Saïdat et al. [12], by means of the normal phase HPLC. The obtained results show that the cyclization reaction followed the first-order kinetics in the solid, nematic, and liquid state of compound A. The value of the estimated activation energy was found to be 101.4 kJ mol^−1^. The observation also showed that the retention times became constant when the B/A quotient reached 5, i.e., the decomposition yield of the A compound was equal to 83 % [12]. The retention values of polyaromatic hydrocarbons and their derivatives, volatile aroma compounds, *cis* and *trans* isomers, and phenols on the A liquid crystal was greater than on the liquid crystal B [12]. The analytical study resulted in the fact that the solid state of B did not completely separate positional and geometric isomers. The B liquid crystal, within its nematic phase, separated decalin and stilbene isomers with the *cis* isomer as the first. With this apart, the separation of the positional isomers of phenols and diethylbenzenes with the more stretched compound having the longest retention time, and the separation of polyaromatic hydrocarbon derivatives illustrated the characteristic separating properties of liquid crystals [12].

The thermotropic liquid crystals with rod-like molecules were tested in the early period of employing liquid crystals as LCSPs, because such compounds were known at that time. Saïdat et al. [13, 14] employed the high performance liquid chromatography (HPLC) technique to determine the values of the kinetic orders, the reaction constants, and the activation energies of the *α*, *β*, iC_4_, and iC_6_ derivatives of the phenyldiazene liquid crystal. The HPLC tests were performed on a 30 cm × 0.4 cm with 5-μm *μ*-Porasil column (Waters) with a mobile phase of *n*-hexane/tetrahydrofuran/acetonitrile—80/18/2 v/v. The kinetics of cyclization reactions of the *α*, *i*C_4_, and *i*C_6_ derivatives of the phenyldiazene liquid crystal were of the first order with activation energies of: 106.4 ± 2.2, 127.4 ± 5.7, and 113.0 ± 3.5 kJ mol^−1^, respectively. These facts apart, the *β*, *i*C_4_, and *i*C_6_ derivatives were employed as the LCSP and showed their excellent separation capabilities for phenolic and polyaromatic compounds in a nematic mesophase. In the separation of geometrical isomers, the known properties of liquid crystals, their rod-like shape, and the ordered arrangement of their molecules have always resulted in the elution of the *cis*-isomer before the trans from the nematic phases [13, 14].

During the synthesising of new liquid crystals, they were also investigated as new prospective chromatographic stationary phases. This concerns the liquid crystals with large molecular masses, including polymers and heterocompounds possessing metal atoms in their molecules. The works describing the thermotropic disc-like and lyotropic liquid crystals as prospective LCSPs have been emerged in the stream of papers describing the general properties of liquid crystals.

The liquid crystals, employed as the chromatographic stationary phases, have the ability to separate the components of mixtures, which have a different molecular structure, i.e., structural isomers, position, and optical, even in the case when the differences in their boiling temperatures, fugitives, and molecular masses are negligibly low. The advantages of the chromatographic applications of liquid crystals as the LCSPs are the relatively wide temperature range of their mesophases which is very useful from the point of view of their applications. The rod-like azo-, azoxy compounds, Schiff’s bases, esters, biphenyl, and terphenyl derivatives generally have useful separation properties. In many cases, the LC temperature ranges can be widened by mixing two or more liquid crystalline compounds. The separation properties of the mixed stationary phases are generally better than the single phases. The widely held opinion is that the nematic phases have better separation properties than the smectic and cholesteric phases, but some of them, not being nematics, also have useful separation properties.

The conventional stationary phases enable the separation of the analytes mixtures, the essence of which is the difference in the analytes vapour pressure and the different solubility of chromatographed substances. Whereas in the case of the liquid crystals, except for the aforementioned factors, the arrangement of molecules forming a mesophase hold a very important function, in employing them as stationary phases. It has also been proved that the separation of the analytes mixtures is not just possible in the mesophase range, but it is also just about possible in its solid and isotropic liquid form.

The diverse mechanisms of the chromatographed substances retentions on the LCSPs depend on the interactions of the ordered liquid crystals structures with different molecular structures of analytes and the acceptor–donor properties of them. A significant property of every stationary phase is closely connected with the bonding and antibonding molecular orbitals, so as a consequence of the acceptor–donor properties is its polarity. As a rule, the LCSPs are the chromatographic phases with moderate polarity. It is one of many important attributes that gives them the possibility to separate analytes with a different polarity.

The LCSPs have not been widely employed in analytical practice, and they are offered by a few firms in a small number of compounds. Nevertheless, they have still been tested. It can be supposed that the seldom use of LCSPs in analytical practice is probably caused by the lack of the universality of employing them. The individual liquid crystalline compounds have different separation properties towards the components of different analytical mixtures. It follows from the interactions of the ordered structure of the LCSP with the different shapes of the molecular orbitals of the analytes molecules, and it is strictly connected with their acceptor–donor properties, namely, with their different interactions with the functional groups of analytes and the cores of the liquid crystals molecules.

Taking the aforementioned deliberations into account, it is necessary to make an attempt to investigate the aim for an answer to the question of whether liquid crystals can be grouped into several classes of compounds similarly interacting, for instance, with derivatives of benzene isomers or/and derivatives of polyaromatic hydrocarbons. After ascertaining which factor has a decisive influence on the retention values of different testing substances with the defined properties of the chemical structure of their molecules, one may propose a liquid crystal molecule, which could be universally employed in chromatographic analysis.

Another impediment in using liquid crystals, such as LCSPs, is that in many cases, the mesophase range is relatively wide, and it is equal to a few dozen degrees. It seldom reached 200°, which is desired in partition chromatography during its employment as the programming temperature mode. The thermal stability of LCSPs is usually worse than the traditional phases employed in analysis, and their volatility is relatively high. In the terms of these properties, better properties other than monomeric liquid crystals are possessed by polymeric ones. The others usually have a higher viscosity and a higher resistance of mass transfer. It is hardly conducive to the efficiency of the column filled with polymeric liquid crystals.

Maybe the aforesaid disadvantages will be diminished. Whether the investigations, which have recently been carried out, leading to improving the LCSPs properties and what is expected of them will come to light under investigation in the future. The profound characteristic and plausible opinion about these investigations is the central subject of our review. The LCSPs investigations carried out over the last 10-year period can be divided into two groups:

(i) the analytical applications of them;
(ii) the physicochemical investigation of LCSPs by means of the IGC technique.

Both investigations mainly encompassed the monomeric LCs, including compounds with heteroatoms in their molecules and also metallomesogenes. Approximately 10 years ago, the new LCSPs group appeared, viz., dendrimers [15]. It is the characteristic feature of the contemporary activities that the majority of the GC tests connected with LCSPs are carried out with the use of the capillary columns.

## Liquid Crystals Stationary Phases in GC

In the chromatographic tests of LCSPs carried out over and within the last decade, a tendency has been conspicuously seen to investigate the interdependence of the spatial arrangements of the LCs molecules and the shapes of the molecules of the separated analytes. The authors of many articles with regard to the analytical applications of the LCSPs draw a comparison between the separation properties of the liquid crystals having different terminal and lateral substitutes.

### Interdependencies Between Chemical Structure of Liquid Crystals and Their Separation Properties

The LCSPs composed of the Schiff’s bases have been investigated for a long time. In the article [16], the separation of the methyl derivatives of phenol on benzylidene-*p*-aminobenzoic acid (442.2–467.2 K) and 4-(*p*-methyl benzylidene)-*p*-aminobenzoic acid (501.2–529.2 K) deposited on Chromosorb W AW and placed in micro-packed columns have been demonstrated. The separation of the mixtures components on these liquid crystals was obtained in the vicinity of their isotropic liquids. Generally, it is assumed that the separation of the mixtures components is better at the beginning of the mesophase range, viz., a few degrees above the melting temperature, when the mutual arrangement of LC molecules is relatively high.

A good separation of the mixture of methyl derivatives of phenol at the end of the mesophases ranges for the LCSPs tested 463.2 and 529.2 K, respectively, have been obtained (see “Appendix: The Characteristics of Liquid Crystals Investigated as Stationary Phases”) [16]. The authors also cited that there is an optimum of the amount of the LCs on the support applied at ca. 20 % in comparison with 15 and 25 %, at which worse separation properties of the LCSPs tested have been observed. The authors gave no explanation of why the results were so strange.

Similar results to those described in the article [16] were described in the article [17]. In this article, the separation properties towards the isomers of cresols on nematics:

4-(Propyloxybenzylidene)-4′-*p*-aminoazobenzene, with the mesophase range 415.2–426.2 K, and 4-(butyloxybenzylidene)-4′-*p*-aminobenzene, for which the mesophase range has not been published, were described. It is only known, that some tests have been performed at a column temperature equal to 449.2 K, within the nematic mesophase. With similarity to the article [16], the optimal amount of the stationary phase was equal to 20 % with respect to the mass support. The selected separation results have been presented at the columns temperatures ranges characteristic for the isotropic liquid.

The investigations described in the articles [16, 17] are the examples of the unreliable description of conducted tests and the results obtained. Our opinion is connected with the factual and editorial point of view. The articles [16–18] have been prepared in different research centres, but the employed methods and chromatographic conditions employed appear to be questionable.

The Schiff’s bases tests are still worth undertaking. The paper, in which the Schiff’s base (4-methoxybenzylideneamino)-4-(2-ethylthio-1,3,4-oxydiazole-5-yl) benzene with oxydiazol groups and $- S -$ bridge was tested as the LCSPs for separation of methyl benzene derivatives, *cis*-decaline, *trans*-decaline, *α*-ionone, and *β*-ionone [19]. The liquid crystal has a relatively narrow nematic mesophase range, from 389.2 to 405.2 K, and it shows differences in the retention times of 1,2,3-trimethylbenzene, 1,2,4-trimethylbenzene, 1,2-dietylbenzene, and 1,3-dietylbenzene that are the compounds with almost similar boiling temperatures. On this LCSP, *α*-ionone [(3*E*)-4-(2,6,6-trimethylcyclohex-2-en-1-yl)but-3-en-2-one] was eluted before the *β*-ionone [(3*E*)-4-(2,6,6-trimethylcyclohex-1-en-1-yl)but-3-en-2-one] and the *cis*-decaline [19] before the *trans*-decaline. They also emphasised that the possibilities of the identification of compounds on the basis of the published retention indices were improved by considering the interface adsorption effects in capillary gas–liquid chromatography [20].

Azoxy compounds being LCs have been tested for a long time as LCSPs. The LCs are usually deposited directly on supports, and the filling is next placed in packed and micro-packed columns The papers, which contain the description of LCs employed for the modification of the materials deposited on the chromatographic supports, have been published in a significant minority. Blokhina et al. deposited 0.5 % of fullerene C_60_ on Chromaton N AW and next different amounts of *p*,*p*′-azoxyphenetol (PAP): 0.05, 0.5, 1.5 % with respect to the mass of Chromaton [21]. In the case of the highest amounts of the LC deposited on the fullerene, the LC phases’ transitions can be observed. The modification of the Chromaton–fulleren system with a slight amount of the liquid crystals enables one to obtain a better separation of polyaromatic hydrocarbons than the results obtained using the system Chromaton–PAP. Among the tested systems containing 0.05, 0.5, and 1.5 % of PAP in the stationary phase, the amount 0.5 % produced the optimal result from the point of view of the separation abilities of the PAP tested. The range of the nematic mesophase of the PAP liquid crystals was as follows 407.7–438.7 K (see “Appendix: The Characteristics of Liquid Crystals Investigated as Stationary Phases”). However, after the PAP deposition on the fullerene of the column, filling would be employed at the wider range of the temperatures than the temperatures describing the mesophase range of the PAP compound, including the temperatures of up to 473.2 K, i.e., higher than the transition temperature to the isotropic liquid.

The other interesting example of the LC application is the LCSP, which was described by Onuchak et al. [22]. They deposited Aerosil-175 (having a particle diameter of 10 nm) on the capillary column wall. The adsorbent layer thickness was 5–10 μm. Thereafter, they deposited 1.64 % of 4-methoxy-4′-ethoxyazoxybenzene (MEAB) with respect to the Aerosil-175 mass (see “Appendix: The Characteristics of Liquid Crystals Investigated as Stationary Phases”).

It has become obvious that the modification of the Aerosil-175 layer with the nematic LC modifier should result in a decrease in the retention factor, *k*, if the retention of adsorbates has been determined to a greater degree by the interaction within the interphase boundaries of the Aerosil–MEAB and the MEAB–vapours phases of the testing substances rather than by the dissolution in the film of the LC. According to the authors, this results in the values of the capacity factor, *k*, of *n*-alkanes (*n*C_6_–*n*C_11_) obtained on the LC-modified column SCOT (SiO_2_ + MEAB) which have been changed from 10 to 20 times smaller than the values obtained on the virgin PLOT/SiO_2_ column [22]. However, for the SCOT column, the capacity for aromatic hydrocarbons changed from 2 to 4 times higher in comparison with that for *n*-alkanes bearing the same number of carbon atoms. This effect has appeared on the *π*-electron interaction between the aromatic systems of adsorbate molecules and the MEAB. Moreover, the low values of the heights equivalent to a theoretical plate, *H*, for the testing substances eluted on the SCOT/SiO_2_ + MEAB filling have been noted [22]. In addition, the separation properties of the SCOT (SiO_2_ + MEAB) and the PLOT/SiO_2_ column fillings have been interpreted on the basis of the separation number parameter, *SN*, that showed the maximum number of peaks that could be recorded in the chromatogram between the peaks of successive homologues of *n*-alkanes. For the following *n*-alkane pairs *n*C_9_–*n*C_10_ and *n*C_10_–*n*C_11_, the *SN* values for the SCOT/(SiO_2_ + MEAB) filling have been equal to 4.0 and 3.6, respectively; however, for the PLOT/SiO_2_, filling those values have been equal to 6.5 and 7.1, respectively [22]. The SCOT/(SiO_2_ + MEAB) has the values of the *para*-*meta* xylenes selectivity, $\alpha_{p/m}$ = 1.07–1.10 within the temperature range 368.2–383.2 K, and the *cis*–*trans* decaline selectivity, $\alpha_{c/t}$ = 1.24, at the temperature of 371.2 K, viz., at the temperature of the beginning of the mesophase existence [22].

Addoun et al. [23] tested, by means of the differential scanning calorimetry, thermomicroscopy, and inverse gas chromatography, three nematic LCs (denoted by LCC_1_, LCC_3_, and LCC_4_—see “Appendix: The Characteristics of Liquid Crystals Investigated as Stationary Phases”) having the same core structure but different lateral substituents. Thus, the authors wished to assess the shape selectivity of the mesogenic LCSPs over the nematic ranges by eluting the same pairs of isomers: *α*-pinene–*β*-pinene, 1,3,5-trimethylpropylbenzene–*n*-propylbenzene, nerol–geraniol, *cis*-decaline–*trans*-decaline, fenchone–camphore, eugenol–isoeugenol at different temperatures. The positional isomers of 2,6-, 2,5-, 2,3-, 3,5-, and 3,4-dimethylphenols were eluted in this order in the nematic state with the three LCSPs, because the hydroxyl functional groups, being a hard base according to Pearson’s principle, were sterically hindered for the intermolecular interactions due to the presence of methyl groups in its neighbourhood, i.e., 2–6 positions. A similar trend was observed with the trimethylphenols, viz., 2,4,6 phenol was eluted before the pair of 2,3,5–2,4,5 phenols, which were co-eluted on each LCSPs tested [23].

The highly toxic 2,6-dichlorophenol and 2,4-dichlorophenol were co-eluted in the phase LCC_1_ (isotropic) and partially resolved in the phase LCC_4_ (isotropic), whereas the aforesaid dichlorophenols were well discriminated in the nematic phase LCC_3_ [23]. Thus, the longer retention in the nematic state resulted in the fact that the more elongated analyte molecules were separated. Whence, the pairs of isomers *m*-chlorophenol–*p*-chlorophenol and *m*-bromophenol–*p*-bromophenol were probably not separated on the three LCSPs. The LCC_4_ column filling revealed better baseline stability, and thus, Addoun et al. [23] could inject the analytes with the higher boiling points, and 2,6-dibromophenol, 3,5-dichlorophenol, and 3,4-dichlorophenol were eluted and separated exactly in this order.

The relatively good separations were obtained for the close-boiling compounds, viz., β-citronellol–nerol and linalyl acetate–borneol in the LCC_1_, whereas in the phase LCC_3_, better separations were obtained for higher boiling analytes, i.e., *cis*-jasmon–*cis*-isoeugenol [23]. Nevertheless, a relatively high selectivity was noticed over the melting points, and the subsequent decreasing of this parameter was noticed by increasing the column temperature. The phases LCC_1_ and LCC_2_ separated the aforesaid isomers better in comparison with the LCC_4_ phase.

The authors of the articles [24–29] show that the comparison of the chromatographic results for the LCSPs with different molecules behaves as though they have an almost similar chemical structure. The afore-mentioned LCs belonged to the azo compounds with different terminal and lateral substitutes and different groups in the aromatic ring [28, 29] and the sulphur compounds with 1,3,4-oxadiazol group in their molecules [30–32].

In [24], it has been stated, that in the LCSP, being a mixture of two nematic liquid crystals LC_*a*_ (with substitutes: 4-pentylcyclohexyl and dodecanoxyl) and LC_*b*_ (with substitutes: 4-ethoxybenzyloxyl and decanoxyl), the second had a decisive influence on the ability to separate a mixture of *meta*- and *para*-cresols, chlorophenols, and bromophenols regarding hard electrodonor properties of the 4-ethoxybenzyloxyl substitute.

The analytical performance of two liquid crystals with the same core substituted with methylbenzyloxy and chlorobenzyloxy chains of medium polarity (denoted by 3-CH_3_ and 3-Cl, respectively) was checked [25]. The range of the nematic phase depended slightly on the relative positions of the lateral aromatic branches (see “Appendix: The Characteristics of Liquid Crystals Investigated as Stationary Phases”). Apart from this fact, the aforesaid liquid crystals behaved as convenient stationary phases for the separation of analytes, such as aromatic compounds, polyaromatic hydrocarbons, phenols, and volatile aroma compounds. According to the authors, the 3-Cl stationary phase enabled a better separation of some positional polyacromatic hydrocarbon isomers and volatile aroma compounds in comparison with the 3-CH_3_ filling.

Relatively good separations of mixtures components were obtained within the nematic phase. The number of theoretical plates with respect to the column filling length in the nematic mesophase is slightly higher than in the solid [19, 26]. The number of theoretical plates for the LCSP being in isotropic liquid or in solid is sufficient enough to obtain the useful separations of mixtures components after all [19]. However, the detailed explanation of the separation mechanisms on the LCSP being solid is still difficult to obtain an unambiguous explanation.

The comparison of the LCSPs differing with one functional group in their molecules is the most correct behaviour, because in such a case, it is possible to assess the influence of the substitute properties in the mesophase range and its separation properties. The evaluation of the causes of change of the LCs properties after the replacement of two or more numbers of groups is ambiguous, if barely possible. Whence, it is difficult to estimate what the influence of particular functional groups on the LC properties.

Benalia et al. [30–33] have described in detail the influence of the chemical structure of three nematic LCs on their separation properties towards about eighty analytes belonging to the different groups of chemical compounds. These LCs have the same basic structure with different substituents, namely: 5-[4-(4-methoxyphenylazo)phenyl]-2-butylthio-1,3,4-oxadiazole—Phase I, 5-[4-(4-butyryloxyphenylazo)phenyl]-2-butylthio-1,3,4-oxadiazole—Phase II, and 5-[4-(4-propoxyphenylazo)phenyl]-2-butylthio-1,3,4-oxadiazole—Phase III (see “Appendix: The Characteristics of Liquid Crystals Investigated as Stationary Phases”). It has been shown that the separation effect of analytes significantly depended on both the LC chemical structure and the chemical structure of the chromatographed substances. The separation effect has also depended on the polarity of the functional groups of mutually interacting molecules with each other and one another during their elution on the LCSPs being solid or liquids characterised by anisotropic properties. The hydroxyl group substituted in the alkoxyl substitute of formylazobenzenes molecules causes a disadvantageous effect on the separation properties.

With regard to the phenol derivatives (also having electron-donor properties), 2,6-dimethylphenol was less retained on the three columns, whereas in the II phase, 3,5-dimethylphenol was retained more, and 2,4-dimethylphenol and 3,5-dimethylphenol were not separated. The retention time of 2,3,5-trimethylphenol was greater than that of 2,4,5-trimethylphenol in the I and III phases, while the former was eluted first in the II phase [30]. Xylene isomers were not separated on the three aforesaid LCSPs. However, the *ortho* isomer was more retained in the phases I and III. As expected, the retention times increased with the number of methyl substituents, as for xylene, trimethylbenzene, tetramethylbenzene, and hexamethylbenzene, so in the same order than their respective boiling points. This apart, it could be noticed that 1,2,3-trimethylbenzene was more retained than 1,2,4-trimethylbenzene, and the following order was observed: *t*-butylbenzene > *i*-butylbenzene > diethylbenzene > tetramethylbenzene in the three phases [30–33]. The main factor in determining their retention is the symmetry of substitution. Benelia et al. observed the 1-methylnaphtalene and 2-methylnaphtalene elution order in the III phase and the inverse order in the I and II phases [30–33]. The observed elution order for dimethylnaphtalenes in the three phases was the same, namely, 2,6-dimethylnaphtalene < 1,6-dimethylnaphtalene < 1,5-dimethylnaphtalene < 2,3-dimethylnaphtalene. The 1,5-dimethyl-naphtalene and 2,3-dimethylnaphtalene isomers were separated only in the phase III.

A significant separation was not noticed between the xylene isomers; however, the separation of the phenol isomers was satisfying on the three LCSPs [30]. Whereas the *o*-ethylphenol was eluted first for cresol and ethylphenol, *o*-phenylphenol was more strongly retained than its *m*- and *p*-isomers. The authors explained such behaviour by the strong interactions between two phenyl rings with the hydroxyl substituent in an *ortho*-position, which distorted the molecule from the planar shape. The retention time lengthened as the number of methyl groups attached to molecules of xylene, trimethylbenzene, tetramethylbenzene, and hexamethylbenzene were in accordance with the increasing values of their boiling temperatures [32, 33]. In this case, the difference in chemical structure of the molecules has no essential influence on the separation effects of the components of the same mixtures [30].

Despite the single liquid crystals, the physicochemical and separation properties of their mixtures have also been investigated. The LCs mixtures usually have wider mesophase ranges. It significantly increases their application properties in comparison with the single compound of LCSP.

**Fig. 1:**
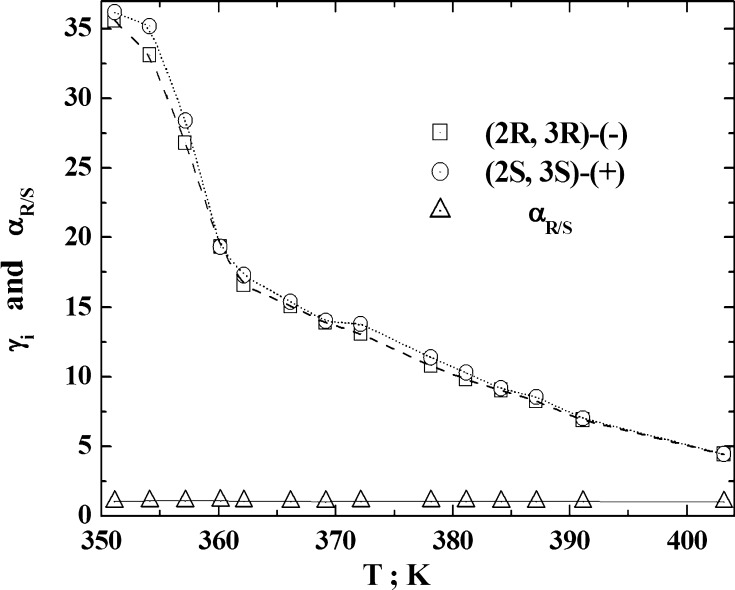
The variation in the values of the activity coefficients of butanediol-2,3 enantiomers and their enantiomers with temperature on the DOBAMBC (prepared using the data taken from: [34])

The separation of the *meta*- and *para*-xylene isomers is still regarded as a classical problem of chromatography. Therefore, the separation ability of the LCSPs is often estimated on the basis of the ability to separate the xylene isomers in these phases. However, the lack of separation of *meta*- and *para*-isomers of xylene has not meant that the LCSP does not possess good separation properties towards the mixtures of other isomers [31–35] (Fig. 1).

Chiral compounds can be separated using chiral stationary phases, including the chiral LC stationary phases. The LCSP, which deserve the chromatographists’ vigilance, is just chiral smectic (S)-2-methylbutyl 4-(4-decyloxybenzylideneamino)cinnamate and 4-(3-hydroxypropyloxy)-4′-formylazobenzene and *β*-cyclodextrin (DOBAMBC and HPOFAB-*β*-CD, respectively—see “Appendix: The Characteristics of Liquid Crystals Investigated as Stationary Phases”) [34, 35]. Alcohols, aldehydes, *n*-alkanes, arenes, and optical isomers have been separated on this LCSP. The attempts of the separations have been performed in $S_{c}^{*}$ and $S_{A}^{*}$ chiral mesophases. Considerable selectivity of the separation of different compounds of different mixtures was obtained, for example, the separation factors for *para*- and *meta*-xylenes were equal to 1.06–1.07, and for enantiomers 1.05–1.09.

According to the authors [35], the introduction of nonmesogenic chiral addition of *β*-CD in an amount of 10 wt% (2.8 mol %) into HPOFAB did not induce the chiral twisted smectic phase, $S_{A}^{*}$. A decrease in the melting point and widening of the temperature range of the existence of the *S*_A_ achiral phase due to the disappearance of the nematic phase was only observed.

Kuvshinova et al. [36] deposited both c.a. 9.95 % 4-butyloxy-4′-formylazobenzene and separately 4-(3-hydroxypropyloxy)-4′-formylazo-benzene on Chromaton N AW. They placed the prepared LCSPs in the columns made of metal and performed the separation of xylenes and cresols within the column temperatures ranging from 366.8 to 385.9 K. The highest selectivity in the separation of xylene isomers, $\alpha = 1.17$, has been obtained for the nematic 4-(3-hydroxypropyloxy)-4′-formylazobenzene. According to the authors, this LCSP could be used for the separation of isomers of high-boiling substances, e.g., *p*- and *m*-methylanisoles and 3,5-lutidine and 3,4-lutidine, however, at the chromatographic conditions more carefully selected than those presented in the article [36].

Since the satisfactory selectivity in the separation of xylene isomers, $\alpha = 1.13$, the nematic 4,4′-methoxyethoxyazoxybenzene was recommended as a preferable LCSP in the chemical analysis [36].

### Copolysiloxanes and Metallomesogenes

Generally, the polymeric LCs are infrequently tested as an LCSP. Therefore, a fewer number of articles on the subject can be observed. Among the polymeric liquid crystals, polysiloxanes are the most often employed as the LCSPs. Finkelmann et al. [37] were the first who employed these kind of stationary phases for the analysis of polycyclic aromatic hydrocarbons (PAHs).

Copolysiloxanes containing [S]-1-(2-naphthyl)ethyl6-[4-(10-undecen-1-yloxy)biphenyl-4′-carbonyloxy]-2-naphthoate mesogenic and 4-biphenyl 4′-allyloxybenzoate mesogenic side groups in the backbone have the cholesteric mesophase within a relatively wide temperature range with the transition temperature to isotropic liquid equal to 510.9 K [38]. These polymers, when employed in capillary columns, had a good separation ability to the PAHs having from two to five aromatic rings in theirs molecules, including benzo[*a*]pyrene and dibenz[*a*,*h*]anthracene. The analysis of 13 PAHs lasted about c.a. 22 min [38].

The LCs with the branched structure of molecules, namely, the dendrimeric LCs have been known for 20 years [15]. These compounds have been tested as the LCSPs, being a single compound or a mixture, for example, the G3 dendrimer with *p*-*n*-pentyloxy-*p*′-cyanobiphenyl [39].

The investigations of dendrimeric LCSPs were at their initial stages, but according to the authors opinion [40, 41], they were very auspicious. Taking the diversity of the branching of different functional groups in molecules into consideration, there existed the possibility of a strong interaction of dendrimers with the support surface, a relatively low viscosity, satisfactory solubility of different chemical compounds, and good mixability with other LCs. Unfortunately, the number of articles giving the examples of the separation of the mixtures compounds on the LCSPs with dendrimers has been relatively low.

The investigations of the soluble and adsorption properties of the dendrimeric LCs with the different chemical structures have been described in articles [39–41]. For example, the physicochemical properties of a single polypropyleneimine dendrimer [39–41] and its mixtures with 4-pentyloxy-4′-biphenylcarbonitrile (5OCB) have been interpreted [39]. On the basis of the Rohrschneider’s constants, the polypropyleneimine dendrimer turned out at a stationary phase with low polarity. It has also been discovered that the support has not disfigured the columnar phase of the dendrimer [42].

In the case of the mixed phases, viz., the columnar phase of the dendrimer and the nematic phase of 5OCB the interaction of any chromatographed substance with the LCSP expressed by means of the thermodynamic quantities are more complicated than for the single columnar phase. It is probably connected with the presence of microdomains of the nematic structure enclosed in the columnar structure of the dendrimer [43].

The increase in the compatibility of *n*-alkanes (*n*C_7_–*n*C_10_) and *n*-alcohols (*n*C_5_–*n*C_8_) with the anisotropic structure of the phases in the phases in the homological series of dendrimers has been demonstrated. *n*-Alkanes had a larger physicochemical affinity with the dendrimeric macromolecules than the *n*-alkohols. *n*-Alkanes non-specifically interact and *n*-alcohols specifically in the chromatographic systems: dendrimer—testing substance [39, 43, 44]. It is probably connected with the creation of hydrogen bonds between the aminolike parts of dendrimers and the hydroxylic groups of *n*-alcohols.

In the articles [40, 41, 43–45], the results of the chromatographic tests performed by employing very short micro-packed columns (40 cm, 1 mm) have been described, being not enough to show that there are sufficiently good separations. The columns efficiencies, employed in these tests, were very low, and the values of the height equivalent to theoretical plates (HETP) were equal up to 8 mm.

Kuvshinova et al. [45] deposited onto Chromaton N-AW (0.40–0.63) c.a. 9.95 wt% of low-polar ‘conventional’ 4,4′-dimethoxyazoxybenzene (LC-1) (Aldrich), high-polar ‘conventional’ 4-propyloxy-4′-cyanoazoxybenzene (LC-2), and supramolecular 4-(2-hydroxyethyloxy)-4′-cyanoazoxybenzene (Ia). The chromatographic results for *p*- and *m*-xylene, 3- and 4-methylanisole, and isomeric lutidins and picolines demonstrated excellent selectivity of the phase based on the Ia phase in relations to high-boiling organic compounds, *α* = 1.75 in the case of 3,4- and 3,5-lutidins, and high selectivity in relations to separation of *para*- and *meta*-xylenes, *α* = 1.12.

The supramolecular 4-(*ω*-hydroxyalkyloxy)-4′-cyanoazoxybenzenes are also widely employed as LCSPs in analytical gas chromatography for separation of structural isomers of organic compounds. For example, for the 4,4′-methoxyethoxyazoxybenzene (MEAB) in the nematic phase, the observed factor of structural selectivity towards *meta*-*para* xylenes separation was equal to 1.13 [45].

The metal-containing liquid crystals, known as metallomesogens, are of much more recent interest, because such materials are expected to have only the intrinsic properties of organic mesogens but also unique properties based on cations of transition elements. The geometry of the complex is often determined by the metal centre incorporated into an organic chelating ligand, and therefore, it can vary from square planar to tetrahedral structures for complexes with a coordination number of four. Hudson and Maitlis [46] suggested that the square planar geometrics of molecules are usually liquid crystals, whereas those of tetrahedral geometric are often not mesomorfic.

The metallomesogenic side-chain polymer—P-LCuSt, with copper carboxylato discotic units in stacks, prepared by the covalent bonding of 14-pentadecenoic acid, stearic acid, and poly(methyl-hydrosiloxane) would be potentially of great interest as LCSPs in partition gas chromatography [47]. The polymer was deposited in the deactivated fused-silica capillaries (15 m × 0.25 mm I.D.). These LCSPs were characterised by shape selectivity and ligand exchange, which are the predominant separation mechanisms in the elution processes. The chromatograms presented by the authors showed that the separations followed the boiling point of phenol derivatives. Nevertheless, there were some exceptions found, namely, 2-nitrophenol (487.2 K) was eluted ahead of the phenol (453.2 K), *o*-methylphenol (464.2 K), *p*-methylphenol (475.2 K), *m*-methylphenol (476.2 K), 2,4-dichlorophenol (483.2 K), and 2,4-dimethylphenol (484.2 K) [47], and *m*-methylphenol (476.2 K) was eluted ahead of the *p*-methylphenol (475.2 K), whereas 2,4-dichlorophenol (483.2 K) was eluted after the 2,4-dimethylphenol (484.2 K), and 2,4,6-trimethylphenol (493.2 K). 4-Bromophenol and 3-methyl-4-chlorophenol having almost the same boiling points (~508.2 K) were baseline separated well [47].

Dialkyl sulphides are soft bases according to the classification of hard and soft acids and bases [48]. Nevertheless, the presence of longer alkyl groups in the analyte molecule might lead one to increase an electron density on the sulphur atom. This apart, it is necessary to add that the sulphur atom in the analytes molecules is able to form association complexes with the stationary phase having an empty orbital at the central metal atom, i.e., cation Ni^2+^ (being a borderline acid according to Pearson’s principle) with an unoccupied orbital *d* [46]. According to the authors, the dialkyl sulphides were quantitated at the optimum conditions, at which a highly reproducible and a relatively low detection limit could be achieved. At these conditions, the separation mechanism might include ligand exchange shape selectivity and polarity interaction [48].

In the case of the metallomesogenic stationary phases besides the ordered structure of mesophase, which maybe has a discotic structure, the presence of the metal atom has a decisive influence on the final separation effect [11]. On account of the metal atom presence in the molecule, the set of interactions between the stationary phase and the testing (chromatographed) substance has the nature of the complexation gas chromatography.

The elution order of sulphides decreased as follows: dimethyl sulphide > allyl methyl sulphide, di-*t*-butyl sulphide, diethyl sulphide, diisopropyl sulphide > diallyl sulphide, di-*t*-butyl sulphide > di-*n*-propyl sulphide > pentamethylene sulphide > di-*sec*-butyl sulphide > di-*n*-butyl sulphide. The authors observed a stronger interaction force of the dialkyl sulphides with the disc-type of matallomesogen due to the fact that symmetrical dialkyl sulphides were more easily soluble in the rod-like structure [48].

In the analytical applications of metallomesogens, the deactivated fused silica capillaries (20 m × 0.25 mm I.D.) have been employed for testing the discotic copper complex-containing the siloxane polymer—P–C_15_CuC_18_ [48]. Similar to the polymeric, the metallomesogenic stationary phases are also fairly sparsely tested; however, some of them have quite good separation properties [11, 48]. For example, it has been proved that the polymeric metallomesogenic stationary phase having copper in its molecule have a good capability of the good separation of organic sulphides [48].

Chou et al. [49] employed the short micro-packed SILCOSTEEL tubes (Restek, Bellefonte, PA, USA), (1.5 or 3 m × 0.04 in. I.D.) filled with disc-type nickelmesogen—*bis*-[1,2-*bis*-(4-undecyloxyphenyl)ethane-1,2-dithiolene]nickel coated on Chromosorb W (5 %, w/w.), which was tested with respect to the separation of the dialkyl sulphides. They elucidated the possible separation effects caused by the employed mesogenic phase under similar chromatographic conditions. The retention time values decreased as follows: dimethyl sulphide > allyl methyl sulphide, di-*t*-butyl sulphide, diethyl sulphide > diisopropyl sulphide > diallyl sulphide > di-*n*-propyl sulphide > pentamethylene sulphide > di-*sec*-butyl sulphide > di-*n*-butyl sulphide [49].

Taking the rule ‘like dissolves like’ into account Chou et al. [49] assumed a stronger interaction force of the dialkyl sulfides with the disc-type metallomesogen than those with the rod-type metallomesogen due to the fact that symmetrical dialkyl sulfides were more easily soluble in the former.

The chiral S_m_C^*^—(S)-4-decyloxy-2-hydroxybenzylidene 4-(2-methylbutoxy)aniline (BPIP) mixed with the poli(methylo-hydrosiloxano-co-dimethylosiloxane) [HMS] copolymer in the ratio 1:1, was tested by Coskun et al. [50]. The mixture BPIP/HMS has a lower clarifying temperature and a narrow range of the S_m_C^*^ mesophase existence in comparison with the pure BPIP component (see “Appendix: The Characteristics of Liquid Crystals Investigated as Stationary Phases”). On the basis of the IGC results, it has been discovered that *n*-hexane and *n*-heptane were good solvents for BPIP and HMS in the temperature range of 363.2–378.2 K [50]. However, the authors did not elucidate why they selected the aforesaid temperature range in their deliberations.

In this context, it is necessary to add that, the articles concerning the synthesis and testing of ionic liquid crystals (ILCs) have begun to appear in scientific literature [51, 52]. In reality, ILCs are the chemical compounds, which have chemical and physical properties for both, i.e.:

(i) ionic liquids (ILs): ionic conductivity, fluidity, and low vapour pressure;
(ii) liquid crystals (LCs) anisotropic physical properties: orientational order of molecules and birefringence.

Fu and Zhang [53] synthesised the poly(amidoamine) dendrimer with ethylenediamine and acrylic acid methyl ester. The obtained ionic liquid crystal polymer has a nematic phase 403.2–453.2 K. The aforesaid authors coated a support with the synthesised material and filled it with a GC column. The packing was employed for the separation of benzene, methylbenzene and *meta*-xylene [53]. Nevertheless, the authors did not report complete information on the chromatographic conditions of the aforesaid separation.

## Chromatographic Investigations of Different Properties of Liquids Crystals

To establish the correlation between the molecule structure and properties of liquid crystals, it is necessary not only to investigate dispersive and acceptor–donor properties of selected compounds but also to follow the variation of these properties in a homogeneous series, since the configuration of both the same or different substituents in the LC molecule may have a significant impact on the anisotropic molecular arrangement.

Neutral, acidic, basic, and amphoteric volatile probes have to be employed to study the dispersive and acceptor–donor properties of LCSPs by the inverse gas chromatography (IGC) technique. Acceptor–donor interactions in IGC can be obtained by subtracting the dispersive interaction from the total interaction.

The aforesaid basic requirements are extremely necessary for the apt description of the retention mechanism in liquid crystalline stationary phases employed in gas chromatography. Gas chromatography is mainly employed as the method of analysis of the chemical components of mixtures. It can also be employed as a method of the testing of column fillings that they could be adsorbents, catalysers, and liquids of low volatility. In this case, it is termed IGC, which enables a characterisation of their interactions with the chromatographed substances, so in other words, the mechanism of chromatographic separations [54, 55]. The testing substances with defined properties enable a characterisation of their interactions with the tested column fillings, so in other words, the mechanism of chromatographic separations. That is why the diversity of chemical groups producing different acceptor–donor properties of liquid crystal molecules opens an increase in the interest of the testing of LCSPs by means of the inverse gas chromatography can be observed. Until recently, the separation properties of the LCSPs mainly tested did not accurately probe the interaction mechanisms of the LC molecules—the molecules of chromatographed substances. Within the last decade, the number of articles, in which the IGC tests encompassing the interactions mechanisms of the LCSP—the chromatographed substance, has significantly increased. Even so in some article titles, the term ‘inverse gas chromatography’ occurs, unfortunately among them, the term ‘reversed-phase gas chromatography’ has also needlessly appeared [56, 57].

### Activity Coefficients

The $\left( {\frac{{a_{1} }}{{w_{1} }}} \right)^{\infty }$ versus $\left( n \right)$ dependencies for *n*-alkanes and *n*-alcohols eluted from the bed of isotropic and mesogen phases of the polypropyleneimine dendrimer can be characterised by the linear plots [57].

The interactions between the testing substances and the liquid crystals, being the components of the LCSPs, are connected with both the chemical structures of the LC molecule and the chromatographed substances as well as with their polarities.

The activity coefficient of the chromatographed substance, $\gamma_{f}^{\infty }$, is also an important parameter, on the basis of which the LCSPs are characterised. The values of the coefficients for the case of the infinite dilution of chromatographed substances, with making allowances for the non-ideality of the mixture carrier gas–vapours of chromatographed substances, can be estimated by employing the following dependency [58]:

1 $$ { \ln }\gamma_{f}^{\infty } = { \ln }\frac{RT}{{Mp_{2}^{\text{o}} V_{g(T)} }} - \frac{{p_{2}^{\text{o}} }}{RT}\left( {B_{22} - V_{2}^{\text{o}} } \right) + \frac{{p_{2}^{\text{o}} j_{3}^{4} }}{RT}\left( {B_{23} - V_{2}^{\infty } } \right), $$

where $M$ is the molar mass of the stationary phase (i.e., liquid crystal); $p_{2}^{\text{o}}$ and $V_{2}^{\text{o}}$ are the vapour pressure and the molar volume of liquid of the testing substance, respectively; $j_{3}^{4} = \frac{3}{4}\left[ {\frac{{\left( {\frac{{p_{\text{i}} }}{{p_{\text{o}} }}} \right)^{4} - 1}}{{\left( {\frac{{p_{\text{i}} }}{{p_{\text{o}} }}} \right)^{3} - 1}}} \right]$ is the correction coefficient; $\frac{{p_{\text{i}} }}{{p_{\text{o}} }}$ is the quotient of pressures, i.e., input and output; $V_{g(T)}$ is the specific retention volume of the chromatographed substance, with reference to the column temperature; $B_{22}$ is the cross virial coefficient between the vapours of the testing substance and the carrier gas, calculated according to the Beattie–Bridgeman’s dependency:

$$ \frac{{B_{22} }}{{V_{\text{C}} }} = 0.461 - 1.158\left( {\frac{{T_{\text{C}} }}{T}} \right) - 0.503\left( {\frac{{T_{\text{C}} }}{T}} \right)^{3} , $$

$B_{23}$ is the cross virial coefficient between the vapours of the testing substance and the carrier gas employed; $V_{2}^{\infty }$ is the partial molar volume of the testing substance; $T$, $T_{\text{C}}$ and $V_{\text{C}}$ are the column and critical temperatures, and the critical volume of the testing substance.

Blokhina et al. [56] calculated the values of the activity coefficient, employing the following dependency:

2 $$ \gamma_{f}^{\infty } = \frac{RT}{{V_{g(T)} p_{2}^{\text{o}} M}} - \frac{{p_{2}^{\text{o}} B_{22} }}{RT}. $$

For the sake of the lack of data coming from systematic tests, it is impossible to compare and interpret the results obtained for Eqs. (1) and (2).

The importance of the interaction effect of the LCSPs with the chromatographed substances is expressed via the thermodynamic functions, such as: the molar differential enthalpy and entropy of solution and adsorption.

To specify the nature of the intermolecular interactions between the studied columnar and isotropic phases of the poly(propyleneimine) dendrimer, the values of the thermodynamic quantities connected with the solution of *n*-alkanes C_5_–C_11_ and *n*-alkanols C_5_–C_8_ at their infinite dilution were determined, which rendered them particularly suitable for characterising the equilibrium interactions within the phases [59]. The values of the activity coefficients expressed in terms of the excess partial molar enthalpy of solution and the excess partial molar entropy of solution, and also the molar enthalpies of vaporisation for the used testing substances were determined. On the basis of the obtained results, the changes of the ${ \ln }\left( {\frac{{a_{1} }}{{w_{1} }}} \right)^{\infty } = f\left( {\frac{1}{T}} \right)$ dependencies were established making allowances for the different values of the molar entropy of solution of the testing substances in the studied poly(propyleneimine) dendrimer [59]:

3 $$ { \ln }\left( {\frac{{a_{1} }}{{w_{1} }}} \right)^{\infty } = { \ln }\frac{273.2 \cdot R}{{V_{g\left( T \right)} \cdot p_{1}^{\text{o}} \cdot M}} - \frac{{p_{1}^{\text{o}} }}{RT}\left( {B_{11} - V_{1} } \right), $$

where $a_{1}$ and $w_{1}$ are the activity coefficients and the weight fraction; $p_{1}^{\text{o}}$ and $V_{1}$ are the vapour pressure and the molar volume of liquid of the testing substance, respectively; and $B_{11}$ is the first virial coefficient for the testing substance.

The thermodynamic characteristics were calculated from the temperature dependencies of the $V_{g\left( T \right)}$ values. The reliability thereof was evidenced by good agreement of all experimental enthalpies of probe evaporation, $\Delta H_{\text{vap}}$, with the enthalpies calculated by the Watson relations, $\Delta H_{\text{vap}}^{*}$ [59]. All the ${ \ln }\left( {\frac{{a_{1} }}{{w_{1} }}} \right)^{\infty } = f\left( {\frac{1}{T}} \right)$ dependencies were linear for both columnar and isotropic phases of dendrimers.

The thermodynamic molar effects of the *n*-alkanes solutions at their infinite dilution were observed during the analysis of the elution of *n*-alkanes on the LCSPs comprising azo compounds with an oxadiazole structure and sulphur atom. For the observed results, the endothermic effect of *n*-alkanes solution in the nematic mesophase was higher than in the isotropic liquid [30–32]. The significance of the effect essentially depended on both the chemical structure of the LC molecule, viz., on the alkyl chain length and the presence of alkoxyl group. The adsorption–desorption of analytes (or testing substances) on the LCSPs belong to a number of processes occurring during the elution of them and therefore it must be carefully elucidated. Blokhina et al. [60] chromatographically tested three *p*-*n*-alkoxycinnamoyloxy-*p*′-cyanoazobenzene LCs, which differed in substitutes attached to the ether bridge. The sorption properties of supercooled smectic phases of homologues of the *p*-*n*-alkoxycinnamoyloxy-*p*’-cyanoazobenzene series were characterised on the basis of universal retention indices. For the tested LCs, the increase in the selectivity of the smectic phases at their supercooling states.

Making numerous attempts of the application of new LCSPs having a diversity of functional groups, which possess different acceptor–donor properties, a growth of interest in investigations of the interactions of the LCSP—the chromatographed probe, is very intelligible.

Nevertheless, it is necessary to realise that the occurrence of ‘structure’ arises first and foremost from the geometry of the molecules, and as such reflects the repulsive forces between them. Thus, the probe molecules–the LC molecules (solute) interactions, will influence the ordering of the LC molecules in the presence of probe molecules, which, in turn, affects the probe density profile around the LC molecules. This apart, any structure of LC molecule can influence the interaction between the dissolved molecules already at large distances.

Whence, at the present, the state-of-art distant progress in the LCSPs investigations without making allowances for the interactions between the LCs molecules and the chromatographed substances with the defined properties could be troublesome. Taking all the subtle results into considerations, one may predict certain rules leading to the synthesis of a more efficient LCSP [61].

### Solubility and Interactions Parameters

Huang et al. [61, 62] analysed the thermodynamic properties of solutes at infinitesimal dilution into the 4,4-bis(heptyloxyl)azoxybenzene being in different mesophases states (BHOAB—see “Appendix: The Characteristics of Liquid Crystals Investigated as Stationary Phases”) using the IGC method. The values of these energies were related to the ‘disorder parameter’ of Flory’s liquid crystal theory [61]. On the basis of the aforesaid parameters, the values of the ‘effective solubility parameters’ for the smectic phase was lower than for the other phases, being determined at lower temperatures and higher phase density. The changes in both one another and each other mutual arrangement of the LC molecules in the mesophases affect the functional group interactions between the LC molecules and the molecules of the testing substances.

The almost linear dependencies for the logarithm of the quotients of the activity coefficients of the employed solutes at two transitions temperatures (S → N: 366.9 K and N → I: 396.2 K) as a function of solubility of solutes for BHOAB, viz., ${ \ln }\frac{{\gamma_{N}^{\infty } }}{{\gamma_{N}^{\infty } }} =  {\text{or}} \ln \frac{{\gamma_{N}^{\infty } }}{{\gamma_{I}^{\infty } }} = f\left( {{\text{Solute Solubility Parameter}};\sqrt {\frac{\text{cal}}{{{\text{cm}}^{ 3} }}} } \right)$ were obtained [61]. For the aforesaid dependencies, one could observe that for the transition to the isotropic phase, the logarithms of the activity coefficients quotient tended to decrease as the solute solubility parameter increased. However, for the S → N transition in BHOAB, the data showed a large positive slope. According to the authors [61], the correlation of the $\ln \frac{{\gamma_{N}^{\infty } }}{{\gamma_{I}^{\infty } }}$ quotient with the solubility parameter seemed to indicate that the mechanism of variation of the reduced transfer free energy for the solutes used revealed primarily from the ‘interaction energy’, rather than from the ‘exclusion volume’ effect. Regarding the organised structure of the LCSP being in the smectic or nematic mesophases, it was possible to postulate, that the LCSPs surfaces created by the ordered LCs molecules were less accessible to the probes molecules than to the supports surfaces. As it is commonly known, in the smectic mesophase, the LCs molecules were organised in layers with the tail part of the LCs molecules forming nano- and microcliffs between the layers where the free volumes would also be formed and just there the probe molecules ‘were found accommodation’ and dispersively interact with the functional groups on the tail tips, named the heptyl groups in the case of the BHOAB molecules [61]. The interaction energy was quantitatively different for the isotropic phase, where the solute molecules might already have access to all parts of the LC molecules, than in the mesophase where the contacting of functional groups between the probes and solvent was significantly impeded, whereas an intermediate situation might occur for the nematic phase, where the LC molecules were aligned in one direction but were relatively free to move along the director axis to interact with the solute molecules. Thus, the solute molecules would have greater freedom to interact with the various parts of the LC molecules, resulting in the value of the interaction energy somewhat similar to the enthalpy of solution in the isotropic state. Whence, the value of the interaction parameter between the molecules of solutes and liquid crystal varied with regard to each other and one another arrangement of the LC molecules in each liquid crystal phase.

The authors [61, 62] estimated the values of the Flory–Huggins interaction parameter, $\chi$, for non-polar and slightly polar molecules of solutes, employing the solubility parameter model. The temperature dependency of the disorder parameter in Flory’s theory of liquid crystals was employed to explain high values of the enthalpy and entropy of solution. Using the aforesaid model the authors employed the following relationship:

4 $$ \chi = V_{1} \frac{{(\delta_{1} - \delta_{\text{LC}} )^{2} }}{RT} + \chi_{s} , $$

where $V_{1}$ is the solute molar volume, $\delta_{1}$ is the solute solubility parameter, $\delta_{\text{LC}}$ is the LC solvent solubility parameter, and $\chi_{\text{s}}$ is the entropy of solution, which has a value nearing 0.3 for the isotropic solution of solutes in polymers.

The term $V_{1} \frac{{(\delta_{1} - \delta_{\text{LC}} )^{2} }}{RT}$ is the enthalpy of the solution.

Regarding the anisotropic solvent molecular structure, there is the possibility that some molecular region (surface) were less accessible than others. Therefore, Huang et al. [61] presented yet another attempt to elucidate their results concerning the determination of the solubility parameter regarding the functional groups involved in the interaction. Their efforts resulted in the values of the ‘effective solubility parameters’ and would no longer represent the values of the cohesive energy for the separating molecules into the ideal gas state.

According to them, the values of the ‘effective solubility parameters’ of liquid crystal phases can be determined employing a sufficient number of the probe solutions with defined properties via the following dependency [61, 62]:

5 $$ \frac{{\delta_{1}^{2} }}{RT} - \frac{\chi }{{V_{1} }} = 2\left( {\frac{{\delta_{\text{LC}} }}{RT}} \right)\delta_{1} - \frac{{\delta_{\text{LC}}^{2} }}{RT} + \frac{{\chi_{\text{s}} }}{{V_{1} }}, $$

where $V_{1}$ is the solute molar volume, $\delta_{1}$ is the solute solubility parameter, $\delta_{\text{LC}}$ is the LC solvent solubility parameter, and $\chi = V_{1} \frac{{(\delta_{1} - \delta_{\text{LC}} )^{2} }}{RT} + \chi_{\text{s}}$, where $\chi_{\text{s}}$ is the entropy of the solution, which has a value approaching 0.3 for the isotropic solution of solutes in polymers, and determined the values of the $\delta_{\text{LC}}$ parameter employing the $\frac{{\delta_{1}^{2} }}{RT} - \frac{\chi }{{V_{1} }} = f\left( {\delta_{1} } \right)$ equation [61].

Huang et al. [61] obtained the improvement in the fitting of the linear dependencies as the smectic phase changed toward the isotropic phase; however, the obtained data were the most scattered for the smectic phase. The data scattering for this phase were higher at the lower temperatures in comparison to the higher temperatures. The obtained results enabled the suggestion that some specific solution effects caused the interaction parameter to deviate from the predicted trend employing the solubility parameter model alone. Nevertheless, these deviation effects were diminished as the liquid crystal solvent approached the isotropic state. Thus, different interactions between solutes molecules and liquid crystal mesophases showed more selectivity towards solutes.

Analysing the results obtained by Huang et al. [61, 62], an unusual trend could be observed with regard to the temperature dependence of the ‘effective solubility parameters’ of liquid crystals, namely, the values of the solubility parameter of the smectic phases of the BHOAB was lower than the nematic and isotropic phases, despite the lower temperatures range [61]. For isotropic organic liquids, the values of the solubility parameters decreased as the temperature increased because of their volume expansion. The molar volumes of liquid crystals also increased with the temperature increase, whereas for the BHOAB the low-temperature smectic phase actually had the lowest effective solubility parameter [61].

Ocak et al. [63–65] synthesised the (S)-4-(2-methybutoxy)-2-hydroxybenzylidene

4-hexyloxyaniline and 4-[4-(2-ethylhexyloxy)benzoyloxy]benzoic acid (denoted by MBHPIMP, MBHOPIMP, and EBBA, respectively) and tested them by means of polarising microscopy, differential scanning, calorimetry and the IGC technique. In these compounds, the neutral *n*-hexylo group was attached to the benzene ring in MBHPIMP, while in MBHOPIMP, a hydroxyl group being a hard Pearson’s base was attached to the benzene ring.

The specific retention volumes of *n*-octane, *n*-nonane, cyclopentane, cyclohexane, ethyl acetate, *n*-butyl acetate, *iso*-butyl acetate, benzene, toluene, ethylbenzene, and chlorbenzene were determined on the aforesaid LCs. Employing the specific retention volumes, the Flory–Huggins liquid crystal molecule—the testing substance molecule interaction parameter, $\chi_{12}^{\infty }$, the ‘hard core’ liquid crystal molecule—the testing substance molecule interaction parameter, $\chi_{12}^{*}$, and the effective energy parameter, $X_{\text{eff}}$, were determined.

According to the theories of Flory–Huggins and the equation of state, interaction parameters can be defined [63–65]:

6a $$ \chi_{12}^{\infty } = { \ln }\left( {\frac{{273.2Rv_{2} }}{{p_{1}^{\text{o}} V_{g}^{\text{o}} V_{1}^{\text{o}} }}} \right) - \left( {1 - \frac{{V_{1}^{\text{o}} }}{{M_{2} v_{2} }}} \right) - \frac{{p_{1}^{\text{o}} \left( {B_{11} - V_{1}^{\text{o}} } \right)}}{RT}, $$

6b $$ \chi_{12}^{*} = { \ln }\left( {\frac{{273.2Rv_{2}^{*} }}{{p_{1}^{\text{o}} V_{g}^{\text{o}} V_{1}^{*} }}} \right) - \left( {1 - \frac{{V_{1}^{*} }}{{M_{2} v_{2}^{*} }}} \right) - \frac{{p_{1}^{\text{o}} \left( {B_{11} - V_{1}^{\text{o}} } \right)}}{RT}, $$

where $v_{2}$ and $v_{2}^{*}$ are the specific volumes of the liquid crystal, and the specific hard-core volume of the liquid crystal, respectively; $V_{1}^{\text{o}}$ and $V_{1}^{*}$ are the molar volumes of the solvent at temperature *T*, and the molar hard-core volume of the solvent; $p_{1}^{\text{o}}$ is the saturated vapour pressure; $B_{11}$ is the second virial coefficient for the gaseous state; $V_{g}^{\text{o}}$ is the specific retention volume.

The effective exchange energy parameter, $X_{\text{eff}}$, in the equation of solid-state theory is expressed as [63–65]:

7 $$ RT\chi_{12}^{*} = p_{1}^{*} V_{1}^{*} \left[ {3T_{1r} { \ln }\left( {\frac{{\sqrt[3]{{v_{1r} }} - 1}}{{\sqrt[3]{{v_{2r} }} - 1}}} \right) + \frac{1}{{v_{1r} }} - \frac{1}{{v_{2r} }} + \frac{{X_{\text{eff}} }}{{p_{1}^{*} v_{2r} }}} \right] , $$

where $V$, $V^{*}$, and $v_{r}$ are the molar volumes of the liquid at the testing temperature *T*, the characteristic molar volume, $v_{r} = \frac{V}{{V^{*} }}$($v_{1r}$ and $v_{2r}$ are the reduced volumes of the solvent and liquid crystal, respectively); $T_{1r}$ is the reduced temperature of the solvent; $p_{1}^{*}$ is the characteristic pressure.

The values of the aforesaid parameters were analysed regarding the influence of the chemical structure of the LCs molecules [63–65].

### Linear Solvation Energy Relationships

Deliberating the solubility of the testing substances in LCSPs, it is necessary to consider that in some chromatographic systems, the different physical–chemical interactions can occur, namely, electrostatic, hydrophilic, hydrophobic, van der Waals, and the hydrogen bond (being a significant part of the acceptor–donor interactions) [66]. In the case of the elution of cresols and acetates, the specific interactions of hydroxyl and acetate groups with the LCs functional groups can decrease the selectivity of the isomers separations, which could be performed if the separation would only be connected with the differences in the chemical structures of the testing substances molecules.

The intermolecular interactions of the LC molecules with 25 testing substances of different properties (*n*-alkanes, *n*-alcohols, and acetates) were studied in the mesophases, at the temperatures ranging from 343.2 to 443.2 K [66]. The Abraham solvation model was applied for the characterisation of these interactions for the systems: the testing substances—the LCSPs [66, 67]:

8a $$ { \log }k = c + l \cdot { \log }L^{16} + rR + s\pi + a\mathop \sum \nolimits \alpha_{2}^{\text{H}} + b\mathop \sum \nolimits \beta_{2}^{\text{H}} , $$

where *k* is the retention coefficient (in general the magnitude, which depends on the kind of the retention magnitude chosen), *R* is the descriptor of the polarizability, *π* is the descriptor of polarity, *l* represents the cavity formation and dispersion contributions taking part between the LCSP and the probes employed, ${ \log }L^{16}$ is the air–hexadecane partition coefficient, determined at 298.2 K, *a* is the ability of the LCSP to interact as a base in the acceptor–donor interaction through hydrogen bonds with acid probes, $\mathop \sum \nolimits \alpha_{2}^{\text{H}}$ is a descriptor of the hydrogen bond acceptor basicity, which is applied to self-associating compounds when they are used as solvents, *b* is the ability of the LCSP to interact as an acid in the acceptor–donor interaction through hydrogen bonds with basic probes, and $\mathop \sum \nolimits \beta_{2}^{\text{H}}$ is the descriptor of the hydrogen bond acceptor acidities, which are applied to self-associating compounds when they are acting as solvents.

The binomials $rR$–($n/\pi$ electron pair), $s\pi$–(dipoles), $a\mathop \sum \nolimits \alpha_{2}^{\text{H}}$–(hydrogen-bond basicity complexes) and $b\mathop \sum \nolimits \beta_{2}^{\text{H}}$–(hydrogen-bond acidity complexes) and $l \cdot { \log }L^{16}$ in Eq. (8a) quantify the different LCSP–probe interactions occurring during the elution of the probes molecules, if the adsorption of them is omitted. The value of the $l \cdot { \log }L^{16}$ product characterises the non-polar interactions, dispersive forces, and the cavity formation. Thus, the sum $c + l \cdot { \log }L^{16}$ appropriately characterises the interactions of neutral probes as *n*-alkanes on non-polar LCSPs. The four other products of Eq. (8a) characterise the different polar interactions.

It is necessarily to emphasise that the ‘effective’ or ‘summation’ solute hydrogen-bond acidity and basicity ($\mathop \sum \nolimits \alpha_{2}^{\text{H}}  {\text{and}} \mathop \sum \nolimits \beta_{2}^{\text{H}}$) could be taken as the $\alpha_{2}^{T}$ and $\beta_{2}^{T}$ scale [66, 67] for the temperature ranges encompassing the phase transition temperatures for the LCs studied. Taking the Abraham and McGowan suggestions into account, Eq. (8a) can be rewritten in the following form [67, 68]:

8b $$ {\text{SP}} = {\text{SP}}_{\text{o}} + mV + s\pi_{2}^{*} + a \cdot \alpha_{2} + b \cdot \beta_{2} , $$

where ${\text{SP}}$ is the solubility or solvent-dependent property such as the logarithm of the solubility of a series of solutes in a given solvent, ${\text{SP}}_{\text{o}}$ is a constant, $mV$ is the McGowan’s characteristic volume defined in terms of the solute bulk molar volume [68], $s\pi_{2}^{*}$ is a measure of the solute dipolarity through hydrogen bonds with acid probes, $\alpha_{2}$ is the hydrogen-bond acidity, $\beta_{2}$ is the hydrogen bond basicity, and *a, b, m,* and *s* are the coefficients characterising the solvent(s).

On the basis of the temperature dependencies of the Abraham equation coefficients, viz., *m*, *s,a,* and *b,* the liquid crystalline properties of the tested LCs and the DSC results for them were confirmed. It also confirmed that $\pi_{2}^{*}$, $\alpha_{2}$, and $\beta_{2}$ in Eq. (8b), that is the explanatory descriptors (according to Rohrschneider [69]) or descriptors of each probe used are correctly chosen.

IGC is widely employed for the testing of the subtle differences in the thermodynamic properties of LCs molecules, whose molecular structures differ very slightly [24, 25]. The authors of the works showed that the differences in the LCs molecular structures, namely, 4-pentylcyclohexyl group in the LC_a_ compound and 4-ethoxybenzyloxy group in the LC_b_ compound and their equimolar mixture can have a significant impact on their separation properties (see “Appendix: The Characteristics of Liquid Crystals Investigated as Stationary Phases”). The comparative IGC study enabled us to predict the separation of the positional isomers of xylenes, diethylbenzenes, phenols, and their derivatives and cresols [24, 25]. The more elongated molecules, such as anthracene, *p*-bromo- and *p*-chlorophenols, and *p*-cresol, were retained more in the phases; however, *p*- and *m*-bromo- or *p*- and *m*-chlorophenols were not totally separated in the LC_a_ phase. The satisfactory resolution of these isomers was observed in the LC_b_ and LC_a+b_ phases [24, 25].

Liu et al. [47] separated phenol derivatives with different substituents on the columnar metallomesogenic polymer. The substituents, such as Cl^−^, NH_2_^−^, and NO_2_^−^, confered greater polarity on phenol molecule, and CH_3_^−^ substituent caused a lesser polar property of the analytes. The methyl group is a soft base, so the property leads to a more stable complex formation toward the stationary phase for *o*-methylphenol than *m*-methylphenol. They observed that the values of the differences between the enthalpy and entropy for the aforesaid probes on the hexagonal ordered, $D_{{({\text{ho}})}}$, and lamellar ordered, $D_{(L)}$, phases. The $\Delta H_{{(L - {\text{ho}})}} = \Delta H_{{({\text{ho}})}} - \Delta H_{(L)}$ values decreased in the following order 2,4,6-trimethylphenol > *p*-methylphenol > 2,4-dichlorophenol > *o*-methylphenol > phenol > 2-nitrophenol > 2-chlorophenol, which was similar to that of the $\Delta H_{(g - h)}$ values. The $\Delta S_{{(L - {\text{ho}})}}$ values assigned as $\Delta S_{{(g - {\text{ho}})}} - \Delta S_{(g - L)}$ were of a similar order [47].

The $\Delta S_{{(L - {\text{ho)}}}}$ values changed in the same order. In this context, it is necessary to add that the greater the $\Delta H_{{(L - {\text{ho}})}}$ values were connected with the stronger probe—lamellar, $D_{(L)}$, phase interactions. The greater the $\Delta S_{{(L - {\text{ho}})}}$ values were connected that the probes used were more compatible with the ordered lamellar, $D_{(L)}$, phase than with the $D_{{({\text{ho}})}}$ phase. According to the authors, a molecule with greater symmetry and higher basicity would be more accordant with the lamellar phase [47]. The authors determined the values of the Gibbs free energy at the temperature equal to the coexistence temperature of the $D_{(L)}$ and $D_{{({\text{ho}})}}$ phases, i.e., 360.7 K. The $\Delta G_{{(L - {\text{ho}})}}$ negative values were obtained, whereas the $\Delta G_{{(L - {\text{ho}})}}$ value for unsubstituted phenol was the lowest [47].

The temperature dependence of the relative retention $\alpha_{B - A}$ of the isomeric probe pairs (i.e., *B* − *A*: 2-chlorophenol—2-nitrophenol; *o*-methylphenol—phenol; 2,4,6-trimethylphenol—*p*-methylphenol and 2,4-dimethylphenol—2,4,6-trimethylphenol) could be expressed via the hyperbolic expression [47]:

9 $$ { \ln }\alpha_{(B - A)} = \frac{{\Delta (\Delta H^{\text{o}} )_{B - A} }}{RT} - \frac{{\Delta (\Delta S^{\text{o}} )_{B - A} }}{R} , $$

where the $\Delta (\Delta H^{\text{o}} )_{B - A}$ and $\Delta (\Delta S^{\text{o}} )_{B - A}$, i.e., the changes of the enthalpy and entropy of solution, respectively, as the result of the subtraction of the *A* value from the *B* value.

Benalia et al. [31] have characterised the nematic LCs comprising the sulphur atom and 1,3,4-oxadiazol group in its molecule, and having the same basic structure. They calculated the values of the excess molar enthalpies and entropies of solution, and the activity coefficients at infinite dilution for *n*-alkanes C_13_–C_19_. They obtained the non-linear dependencies of the values of the excess molar enthalpies, entropies, and free energies of *n*-alkanes as the function of the number of carbon atoms in their molecule. Thus, the variations of logarithm of the probe activity coefficients at infinite dilution, ${ \ln }\gamma^{\infty }$, became implausible [31].

The mechanism of the separation of the mixtures components has been tested over the column temperature range encompassing the $S_{c}^{*}$ and $S_{A}^{*}$ mesophases and the isotropic phase of (S)-2-methylbutyl 4-(4-decyloxybenzylideneamino)cinnamate (denoted as DOBAMBC) [34]. The standard and excess thermodynamic parameters sorption of 26 volatile compounds of various classes [*n*-alkanes, arenes, aldehydes, monohydroxylic alcohols, (2R,3R)-(−)-butanediol-2,3, (2S,3S)-(+)-butanediol-2,3, camphene(−), and camphene(+)] have been determined. The maximum enantioselectivity was $\alpha_{R/S}$ = 1.096 for ∓butanediols-2,3 at 360.2 K in mesophase $S_{C}^{*}$, and the pronounced enantioselectivity values for camphenes $\alpha_{ - / + }$ = 1.062 at 381.2 K in the mesophase $S_{A}^{*}$. According to the authors [34], the tilted and perpendicular orientations of long molecular axes with respect to the plane of layers of the smectic LC tested had a decisive influence on the mechanism of the chiral recognition of the optical isomers of polar and low-polar probes during the elution process.

The 2-ethylpropyl radical at the end of the LC molecule contains an asymmetric carbon atom, which is the reason of the twisting structure of the smectic mesophases. According to the authors in the $S_{C}^{*}$ phase, each layer is turned through some angle with respect to the preceding layer to form a twisted structure of the LC with a certain helix pitch. Whereas in the $S_{A}^{*}$ smectic phase, the twisting arrangement occurs in the plane of layers. Thus, an increase in the column temperature caused the structural changes in [34]:

(i) the orientation and the translational arrangement of the LC molecules;
(ii) the measurable changes in the helix pitch;
(iii) the mutual arrangement of long molecular axes with respect to the LC molecule—solid support and the interphase: LC molecule—gas phase.

The transition of the solid LC into the $S_{C}^{*}$ mesophase did not cause an essential increase in the $V_{{g(T_{\text{c}} )}}$ values because of the change of the elution mechanism from the dynamic sorption of 26 probe vapours by a chiral smectic liquid crystal, i.e., 2-methylbutyl ester of 4-(4-decyloxybenzylideneamino)-cinnamic acid from the gas phase to the solution of probe vapours in liquid. However, in the $S_{C}^{*}$ mesophase, an increase in the $V_{{g(T_{\text{c}} )}}$ values up to temperatures of ~360.2 K and a subsequent decrease in the $V_{{g(T_{\text{c}} )}}$ values in the temperature range from ~363.2 to ~ 393.2 K were observed [34]. Nevertheless, it was shown that the layered twisted structure of the mesophases of the DOBAMBC system played a key role in the mechanism of chiral recognition of butanedion-2,3 and camphene enantiomers [34], whereas the isotropic phase of the LC tested did not possess such a property. If the vapour pressures of the enantiomers are equal, whence, the value of the separation factor under the gas–liquid chromatography conditions depends on the ratio between their activity coefficients, i.e., $\alpha_{1,2} = \frac{{\gamma_{2}^{\infty } }}{{\gamma_{1}^{\infty } }}$. The changes of $\alpha_{1,2}  {\text{vs}} . { } t$ are shown in Fig. 1 prepared on the basis of the Onuchak et al. data [34].

The aforesaid authors indicated that the LC melting substantially increased the $V_{{g(T_{\text{c}} )}}$ values for arenes. This was more than likely related to a more planar structure of the arenes molecules and the possibility of their incorporation into the structure of the $S_{C}^{*}$ tilted smectic [34]. This apart, the predominant influence of the probe molecule shapes on their ability to dissolve in the DOBAMBC mesophases enlarged the *para*-xylene retention in comparison to the magnitude of the *meta*-xylene isomer.

The enantioselectivity of the HPOFAB-*β*-CD binary phase was considered in relation to optical isomers of limonene, camphene, butanediol-2,3, and menthol [35]. Moreover, the enantioselectivity in relation to isomers of bicyclic camphene was observed in all phases the HPOFAB-*β*-CD adsorbent with increasing retention of the (+)-isomer [35].

Blokhina et al. [56] tested the chromatographic behaviour of 2-methylpropanol-1, butanol-1, and butanol-2 as probes, on cholesteryl tridecylate deposited on Chromaton NAW in the amount of 5 wt%. The choice of the probes was defined by the chemical structures of the alcohol molecules having the hydroxyl group in a variety of positions and having conveniently measurable saturated pressure in the temperature range of the existence of smectic, cholesteric, and isotropic phases of cholesteryl tridecylate.

The IGC technique, when employed to the liquid crystal stationary phases, may produce implausible results even when the precision of experimental data and the correctness of their interpretation are accomplished. Therefore, the authors [56] checked, among other things, the input of the LC amount on the $V_{{g(T_{\text{c}} )}}$ data only for the 5 % content, but they stated that those changes of the $V_{{g(T_{\text{c}} )}}$ and the activity coefficients at infinite dilution of butanol isomers values for the higher amounts of more than 5 % of the LC tested were insignificant and they fell within the experimental error, which was indicative of sorption in the bulk of the LCSP tested for them [56]. Despite this, the authors had the courage of their convictions towards their results; however, the support impact on the LCSP properties at such low coverage of its non-acid washed surface (i.e., Chromaton N AW) seems to be taken into consideration [34].

Unfortunately, the similar misgivings about the results published by Kuvshinova et al. bring to our mind other misgivings [36, 45]. Admittedly, the authors separately deposited c.a. 9.95 % of 4-butyloxy-4′-formylazobenzene, 4-(3-hydroxypropyloxy)-4′-formylazo-benzene, 4,4′-dimethoxyazoxybenzene, 4-propyloxy-4′-cyanoazoxybenzene, and 4-(2-hydroxyethyloxy)-4′-cyanoazoxybenzene on the Chromaton N AW. After this, they placed the prepared LCSPs in the columns made of metal and analysed the elution of xylenes and cresols within the column, temperatures ranged from 366.8 to 385.9 K. According to the authors, the structural selectivity of the separation of the weak-donor xylenes was given by the entropy contribution, which was caused by the considerable differences in the steric effect exhibited in the dissolution of xylenes, whose aliphatic substituents were bonded by hydrogen bonds with the LCs tested [36, 45]. The trends comparable with the previously described were also observed in the dissolution of electron-donor lutidine isomers, which was obviously caused by the impact of proton-donor hydroxyl groups on the specific cohesion interactions of the LCSPs [36, 45].

The presence of hydroxyl groups in the molecules of the *p*-cresol and *m*-cresol assured the facts of:

(i) the possibility of significant interaction with both the active terminal substituents of LCs;
(ii) the equalisation of the values of the thermodynamic parameters of adsorption;
(iii) the reduction of the structural selectivity of the LCSPs (i.e., $\alpha_{\text{I}}$ = 1.13 and $\alpha_{\text{II}}$ = 1.17 [35].

Nevertheless, the impact of Chromaton NAW and the column material on the equalisation of the values of the thermodynamic parameters and the reduction of the structural selectivity was not discussed by the authors. Admittedly, the authors wanted to persuade the readers that they checked the amount influence of the deposited stationary phases on the results obtained; nevertheless, they did not present and discuss the errors committed during the determination of the thermodynamic values. Moreover, the peculiarities connected with the specific interactions of the electron-donating sorbate, and the proton-donating terminal group of the LCs is not taken into account in the case of sufficiently basic testing substances [36, 45].

The measurements of the physicochemical quantities characterising the interaction of the chromatographed substance with the LC were performed during the heating and cooling of the capillary column of 30 m × 0.25 mm I.D. [70]. The LC with intermediate polarity was dynamically deposited on the column from a 10 % solution in dichloromethane.

The IGC tests consisted of the determination of the retention times, related retention coefficients, and the values of the enthalpy solution of six trimethylbenzenes, *cis*- and *trans*-decalin, tetramethylbenzene, allylbenzene, and dimethyl-1,3-benzene at different temperatures. The polarising microscopy and the DSC tests were performed aside from the IGC tests. According to the authors [70], the DSC curves for the LC acquired during the first cycle showed a solid-isotropic transition at 377.4 K. When the compound was cooled down to room temperature at 10 K min^−1^, the nematic phase appeared at 345 K [70]. However, the DSC curves during the heating and cooling were acquired with different temperature change rates. The cooling which was started from melting at 393.2 K down to 263.2 K showed that the nematic transition disappeared and the LC tested crystallised during cooling at a slow rate. In addition, the nematic phase showed that recrystallisation of the tested compound increased when the annealing temperature decreased [70]. It should be noted that the IGC results confirmed the nematic transition on the cooling and isotropic transition on heating.

When low-molecular weight LCs are bonded by means of polymeric siloxane, it is possible to obtain polymeric LCSPs, which can be applied in GC and LC also. The mechanism of separation of the mixture components on the polymeric LCSPs is rarely tested. The method of testing of the mechanism is done via the comparison of the thermodynamic properties of low-molecular LCs and polymers, which included them. Attention was especially paid to the properties of the overcooled mesophase. It is interesting, because the properties of the overcooled phases are seldom tested recently.

The thermodynamic properties of the low-molecular LCs: 4-(*n*-hexyloxy)-4′-cyanobiphenyl (HCB) with the nematic phase ranged from 330.2 to 349.2 K and 4-(*n*-octyloxy)-4′-cyanobiphenyl (OCB) with the $S_{A}$ smectic phase ranged from 328.2 to 340.2 K and the nematic phase ranged from 340.2 to 349.2 K were compared with the properties of the poly(dimethyl-co-methyl(4-cyanobiphenoxy)butyl siloxane, which had 40 repeated mers and the smectic mesophase between 269.2 to 352.2 K [71]. The HCB and OCB were also bonded with poly(dimethyl siloxane), which was a skeleton of liquid crystalline polymer. The obtained polymers demonstrated the presence of the mesophase which was equivalent to the kind of mesophase of the low-molecular LC.

The interaction tests of the low-molecular and polymeric LCs with the chromatographed substances were carried out using *n*-alkanes, benzene, cyclohexane, toluene, *p*-, *m*- and *o*-xylenes, 2-methylhexane, 3-methylhexane, 2,3-dimethylpentane, 2,4-dimethylpentane, and 2,2,3-trimethylbutane. The values of the infinite dilution Flory–Huggins interaction parameters, $\chi$, as well as the hard-core interactions parameters, $\chi^{*}$, and the energy exchange parameters, $\chi_{12}$, were correlated with the structural features of the LCs tested and enthalpic and entropic contribution to these interactions [71]. The analysis of the obtained results led to the conclusion that during the elution of non-polar hydrocarbons, the molecular structures of the LCSPs are important. In the case of polymeric SPs bonded with low-molecular liquid crystals, the polymer impact on the separation process is very low [71].

The thermodynamics of UMBPIMP were investigated to understand in depth the interactions between the liquid crystal and different probes for its further exploitation. Therefore, the Flory–Huggins polymer–solvent interaction, $\chi_{12}^{\infty }$, hard-core polymer–solvent interaction, $\chi_{12}^{*}$, and exchange parameters, such as effective energy, $\chi_{\text{eff}}$, enthalpy $\chi_{12}$, and entropy, $\chi_{\text{s}}$, for the probes employed were determined and discussed [61, 62, 72].

The couverture of the surface of the non-specific adsorbent, deposited on plain support as the LC monolayer or polylayer, may enable the elucidation of the selectivity mechanism of the LC. The tests to such an objective was carried out by the deposition of *p*,*p*′-azoxyphenetol (PAP) on the C_60_ fullerene deposited in different amounts on the Chromaton N AW support. The amounts of the PAP deposited were equal to 0.05, 0.5, and 1.5 % with respect to the mass of Chromaton.

On the LCSP prepared in such a way, *n*-alcohols, *para*- and *meta*-xylenes, 3,4- and 3,5-lutidine, *α*- and *β*-naphtols, phenantrene, and antracene were eluted. The adsorption of the aforesaid substances on the stationary phases containing different amounts of PAP was tested on the basis of the values of the specific retention volumes and the enthalpies of adsorption. The analysis of the obtained results leads to the conclusion that the molecules of the testing substances containing hydroxyl groups create the hydrogen bonds of these groups with the oxygen atom of the azoxy group of the LC molecule. The created hydrogen groups play the important role in the interaction pair chromatographed substance–liquid crystal.

### Acceptor–Donor Properties

Ocak and his coworkers tested chromatographically the acceptor–donor properties of 4-[4-(2-ethylhexyloxy)benzoyloxy]benzoic acid (EBBA), 4-bromo-1,3-phenylene-bis[4-[4′-(10-undecyloxy)-benzyloxy]]benzoate (BPUBB), (S)-4(undec-10-enyloxy)-2-hydroxybenzylidene-4-(2-methylbutoxy)aniline (UMBPIMP), (S)-4-(dodecyloxy)-2-hydroxybenzylidene-4-(2-methylbutoxy)aniline (SALC)—Schiff-base, and (S)-4-(5,5,6,6,7,7,8,8,9,9,10,10,10-tridecafluorodecyloxy-2-hydroxybenzylidene-4-(2-methylbutoxy)aniline (SFSALC) [65, 73–75].

The values of the dispersive component of the surface free energy, $\gamma_{\text{S}}^{\text{D}}$, the specific free energy of adsorption, $\Delta G_{\text{A}}^{\text{s}}$, the enthalpy and entropy of adsorption, $\Delta H_{\text{A}}^{\text{s}}$, $\Delta S_{\text{A}}^{\text{s}}$, of neutral, acidic, basic, and amphoteric probes on the LC studied at 308.2 and 333.2 K, viz., at its solid [65]. The $\Delta H_{\text{A}}^{\text{s}}$ values were correlated with the acceptor and the donor numbers of the probes employed to quantify the acidic $K_{A}$ and the basic $K_{B}$ parameters of the LC surface. The values of $K_{A}$ and $K_{B}$ quantities determined were equal to 0.0497 ± 0.0025 and 0.0425 ± 0.0041, respectively. Whence, the EBBA surface was acidic in nature [65].

The values of the dispersive component of the surface free energy, $\gamma_{\text{s}}^{\text{D}}$, the specific free energy of adsorption, $\Delta G_{\text{ads}}^{\text{SP}}$, the enthalpy of adsorption, $\Delta H_{\text{ads}}^{\text{SP}}$, the entropy of adsorption, $\Delta S_{\text{ads}}^{\text{SP}}$, of neutral, acidic, basic, and amphoteric testing substances were employed for the testing of both LCs. The $\Delta H_{\text{ads}}^{\text{SP}}$ magnitudes were correlated with Gutmann’s modified acceptor, ${\text{AN}}^{*}$, and donor, DN, numbers for the testing substances with the aim to quantify the values of the acid, $K_{A}$, and the basic, $K_{D}$, parameters of the LCSPs surfaces.

The specific case of rod-like LCs constitutes ‘bent-core’ LCs. One of them is 4-bromo-1,3-phenylene-bis[4-[4′-(10-undecyloxy)-benzyloxy]]benzoate with the nematic mesophase ranged from 338.4 to 382.3 K [72]. The tests of the acceptor–donor properties of the LCSP with BPUBB compound were performed within the column temperature range 308.2–333.2 K, by employing non-polar, polar, and amphoteric testing substances. The IGC tests were performed at the infinite dilution conditions of adsorbates. The dispersive component of the surface free energy, $\gamma_{\text{s}}^{\text{D}}$, for the LC tested was determined for nonpolar organics. The values of the specific enthalpy of adsorption, $\Delta H_{\text{ads}}^{\text{SP}}$, were determined and correlated with both the donor and the acceptor numbers of the probes used to quantify the acid $K_{A}$ and basic $K_{D}$ parameters of the LCSP surface [72]. The values of the $K_{A}$ and $K_{D}$ parameters were found to be 0.033 and 0.316, respectively, and the ratio of the $\frac{{K_{D} }}{{K_{A} }}$ quotient between 35 and 60 °C was equal to 9.63; it meant that the BPUBB surface exhibited a basic character.

The acceptor–donor and surface characteristic of SALC and SFSALC liquid crystals were performed on the IGC results. The values of the $K_{A}$ and $K_{D}$ parameters for the SALC compound were found to be equal to $K_{A} \cong$ 0.03 and $K_{D} \cong$ 0.13 $( {\frac{{K_{D} }}{{K_{A} }} \cong 4.3} )$ [74]. The values of the $K_{A}$ and $K_{D}$ parameters for the SFSALC compound were found to the Fowkes approach ($K_{A} \cong$ 0.034 and $K_{D} \cong$ 0.44), to the Dong approach ($K_{A} \cong$ 0.16 and

$K_{D} \cong$ 0.69), regarding decadic log of vapour pressure ($K_{A} \cong$ 0.05 and $K_{D} \cong$ 0.23), the boiling temperature ($K_{A} \cong$ 0.03 and $K_{D} \cong$ 0.23), $( {4.3 < \frac{{K_{D} }}{{K_{A} }} < 12.9} )$ [75]. Regarding these values, the surface of the SFSALC exhibited a more basic character than that of SALC between 303 and 323 K [74, 75]. According to the aforementioned authors, this can be explained by the high electronegativity of the fluorine atoms forming the hard C–F dipoles which are strong polar, hydrophobic in nature, and weakly polarisable [75, 76].

Some LCs exhibit the mesophase only on cooling, and they are called monotropic liquid crystals. The monotropic behaviour of LCs is usually tested by means of the differential scanning calorimetry (DSC), polarising optical microscopy and X-ray diffractometry. Ammar-Khodja et al. [70] chromatographically tested the LC with a crown ether bonded in the side of its molecule employing the following probes, i.e., dimethyl-1,3-benzene, trimethyl-1,2,3-benzene, trimethyl-1,2,4-benzene, trimethyl-1,3,5-benzene, tetramethyl-1,2,3,4-benzene, allylbenzene, *cis*-decalin, and *trans*-decalin. They obtained the linear dependencies of the retention factor versus the reciprocal column temperature in two temperature ranges intersecting at the melting point of the LC tested for all the probes employed. Nevertheless, the effect of the ordered structure of the LCSP tested on obtaining the separation of probes mixtures was greater than the effect of the polarity of this LC tested [70].

The thermodynamic properties of the solution of *n*-alkanes, *n*-alkohols, *m*-, *p*- and *o*-xylenes, 3,4-lutidine, 3,5-lutidine, *p*- and *m*-toluidines, *α*-naphthalene, and *β*-methylnaphthalene in the mesoporous and isotropic phases of the G1–G4 poli(propylenoimino) dendrimers were determined by means of the IGC technique [43, 59]. The dendrimers were deposited onto Chromaton NAW-DMCS in amounts equal to 10 ± 0.2 %. According to the authors, the G1–G4 dendrimers have been chosen for two reasons [43, 59]:

- First, their molecules have bush-like alkyl-groups, which are known to determine the sorption properties of hyper-branched polymers.
- Second, their LC phases exist within the closely overlapping temperature ranges.

Blokhina et al. [59] observed that the parameters of the probe retention were sensitive to the phase transitions of the LC phases. The ${ \ln }V_{g(T)} = f\left( {\frac{1}{T}} \right)$ plot clearly showed that there were four types of the phase state: crystalline, first columnar, second columnar, and isotropic liquid. The temperatures of the melting, transition between the two LC phases and clearing could be recognised in the changes of the retention parameters plot. The temperatures of the phase transitions determined by IGC were in good correlations with those of the DSC and thermomicroscopy techniques. The aforesaid authors classified the poly(propyleneimine) dendrimers G1–G3 on the basis of the standard Rohrschneder’s solutes eluted at 373.2 K [40]. According to the existing classification, the aforesaid stationary phases under the IGC tests can be regarded as low-polar [43, 77].

In all cases, the ${ \ln }V_{g\left( T \right)} = f\left( {\frac{1}{T}} \right)$ plots exhibited a common feature, namely, their slopes suffered abrupt changes at the phase transition temperatures. That meant that the local structure of the LCSP, in which the molecules of the testing substances were incorporated, suffered changes upon the phase transitions. The highest sorption capacity, connected with the local structure of the LCSP, and whence, a free volume available for the probes molecules were observed for the G2 dendrimer [59]. For all the probes, these changes were less pronounced in the case of the G3 dendrimer [59]. It was probably connected with the low phase transition enthalpy for this dendrimer. The G4 dendrimer did not form the LC phase; however, the $V_{g\left( T \right)}$ values for its isotropic phase were lower than ones for the G3 dendrimer. Thus, the molecular structure of the dendrimers restricted only the sorption of aromatic probes, and the sterically flexible *n*-alkanes fitted themselves into the brush-like shell [59].

As is commonly known, dendrimers are also classified by generation, which refers to the number of repeated branching cycles that are performed during its synthesis. This parameter (or regularity) can also be used to characterise the thermodynamic properties of dendrimers [78, 79]. The relationships between the activity coefficients and generation number indicated that the lowest $\left( {\frac{{a_{1} }}{{w_{1} }}} \right)^{\infty }$ values, and therefore, the highest thermodynamic compatibility of the probes with both liquid phases was observed for the G2 dendrimer. However, the higher activity coefficients for the G3 and G4 dendrimers indicated a trend of decreasing compatibility with the increase in the generation number. Only the G1 dendrimer did not follow this trend. This apart, the differences between the partial molar enthalpies, $\Delta H_{\text{s}}$, for the xylenes and methylnaphtalenes employed in that study could be attributed to geometrical anisotropy being in advantage to the alignment of the probe molecules with the terminal alkyl groups. According to the authors [59], the aforesaid alignment was expected to be more efficient for *p*-xylene and *β*-methylnaphthalene than for *m*-xylene and *α*-methylnaphthalene, respectively.

In the case of the isotropic phase, the differences between the $\Delta H_{\text{s}}$ magnitudes for isomeric xylenes and naphthalenes indicated the aforesaid alignment in the isotropic phase. The higher negative $\Delta H_{\text{s}}$ and $\Delta S_{\text{s}}$ magnitudes for methyl naphthalenes as compared with the $\Delta H_{\text{s}}$ and $\Delta S_{\text{s}}$ ones for xylenes were attributed to the higher polarizability of the naphthalenes employed, i.e., two-ring molecules.

According to the authors [59], the retention data for isomeric hydrocarbons and amines indicated that the isomers were eluted in accordance with their boiling temperatures, whereas the elution order was only inverted for *p*- and *m*-xylenes. The more efficient dissolution of *p*-xylene in the G1–G4 dendrimeric SPs was probably connected with the intermolecular alignment resulting in the strengthening of the dispersion probe–solvent interactions. The selectivity of the tested LC phases increased in the following sequence: methyl-naphthalenes < xylenes < lutidines < toluidines. The increasing selectivity of the G1–G3 dendrimers could be observed for both columnar and isotropic phases [69].

The results of the chromatographic tests performed by Blokhina et al. [80] proved that in two-component mixtures, dendrimer macromolecule—*n*-alcohol, a declining ability of *n*-alcohols to the specific interactions significantly influenced the entire intensity of the adsorbate–adsorbent interactions, as well as the decrease in the differential molar enthalpy solution.

**Fig. 2:**
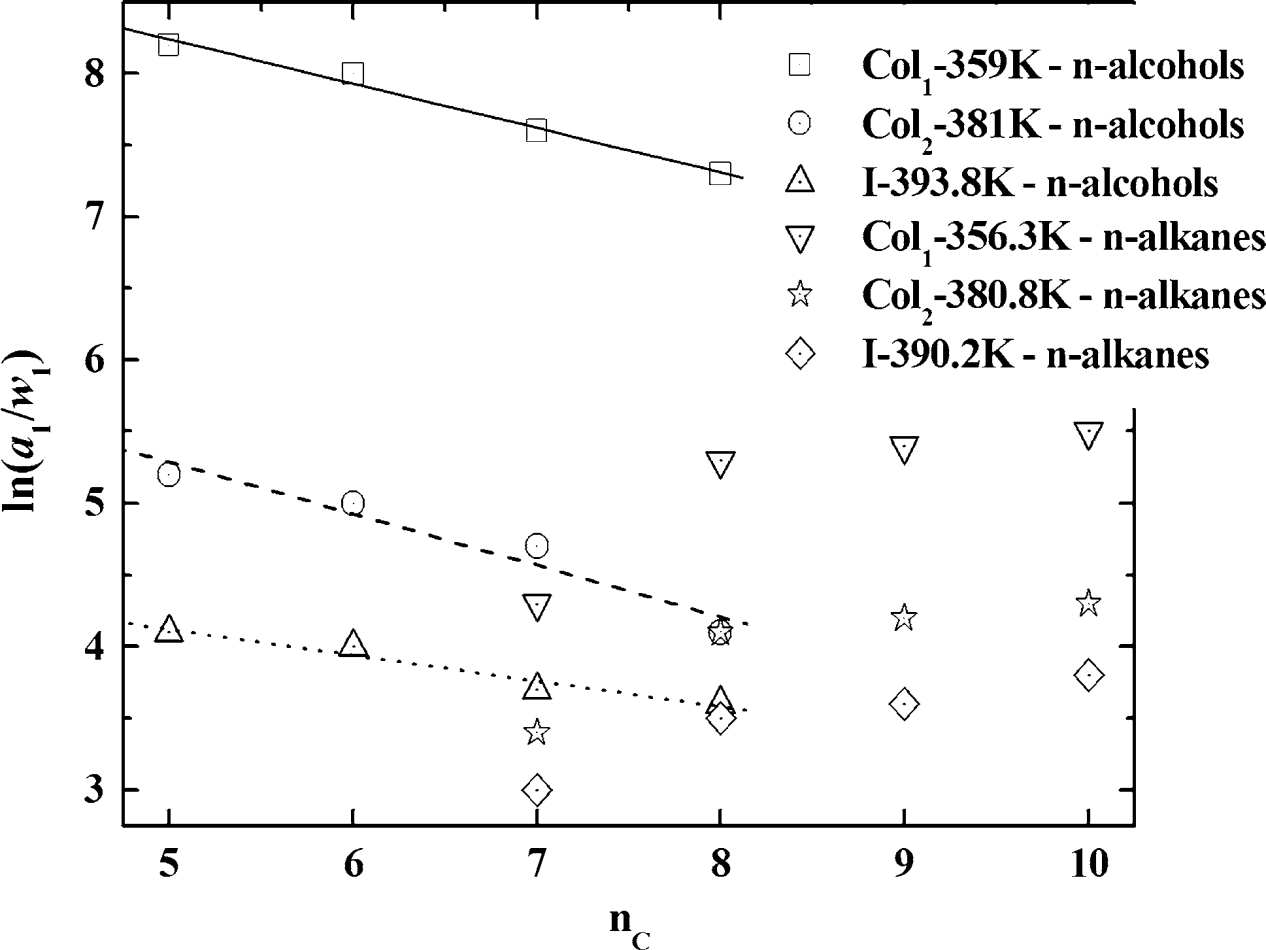
The variation in the $\ln \left( {\frac{{\alpha_{1} }}{{w_{1} }}} \right)$ ratio versus number of carbon atoms in molecule $n_{C}$ for *n*-alkanes C_*n*_H_2*n*+2_ (C_7_–C_8_) and *n*-alkanols C_*n*_H_2*n+1*_OH (C_7_–C_8_) eluted on liquid-crystalline propyleneimine dendrimer (prepared on the data presented in the article: [57])

To explain the influence of the values of the specific contribution of the intermolecular interactions of the tested dendrimers with *n*-alkanes, they were compared with the enthalpy of solution, $\Delta H_{\text{sp}}^{\infty }$, of *n*-alcohols C_5_–C_8_. In all the phases of the poly(propylenimine) dendrimer, the $\Delta H_{\text{sp}}^{\infty }$ values decreased with increase in the –CH_2_– number in the chain of alcohol solved in the mesophase. It was caused by the hydrophobic effect of the interaction of the aliphatic probe and the ≡CH groups of the alkyl substituents at the periphery of the dendrimeric macromolecules [57]. In the case of alcohols, the synenergetic effect of the hydrophilic (hydrogen bond) and hydrophobic parts of probes with the dendrimer macromolecules was observed. The more negative $\Delta H_{\text{sp}}^{\infty }$ magnitudes for polar probes, than for *n*-alkanes, suggested the possibility of the existence of the specific interactions of them with the dendrimer molecules [57]. The $\Delta H^{\infty }$ magnitudes being the contribution of the specific interaction energy to the total value of the dissolution enthalpy of *n*-alcohols were determined from the difference between the $\Delta H_{\text{sp}}^{\infty }$ values experimentally determined for *n*-alcohols, and the values of the non-specific contribution (dispersive) component, $\Delta H_{\text{nsp}}^{\infty }$, determined on the basis of the dependency between enthalpy and polarizability, $\left( { - \Delta H} \right)_{\text{nsp}}^{\infty } = f\left( \alpha \right)$, for *n*-alkanes (Fig. 2).

The energy of the creation of hydrogen bonds (specific) between tertiary amines and phenol groups in the ‘rigid core’ of the dendrimer and the –OH groups of the alcohol molecules comprised a relatively low part of the total energy of interactions, being equal to −16.5 kJ mol^−1^. The value of the Kovač retention index for *n*-heptanol eluted from the dendrimer bed was equal to 974 and it was comparable for the same parameter achieved on phenyl-silicone phases of medium polarity [57]. Thus, the dendrimeric macromolecules are absorbents having the reactive amino groups attached to the ‘rigid core’, which are isolated by the alciloterminal groups, and therefore, they have no influence on the absorption properties of the dendrimer.

The polymeric chemically bonded liquid crystal stationary phase for HPLC, i.e., poly-*γ*-benzyl(*L*-glutamates), has enabled the satisfactory separation of polynuclear aromatic hydrocarbons with 1 to 8 rings in less than 15 min by the use of a linear gradient from 0 to 20 % of chloroform in n-hexane [19].

## Liquid Crystal-Based Stationary Phases for HPLC

Already in 2002, Gritti and Félix [81] meticulously studied the problem of employing liquid crystals, from low- to high-molecular weight, in the HPLC technique.

Liquid crystal stationary phases are not only employed as the column fillings for GC but also as the column fillings for high performance liquid chromatography (HPLC). To obtain a column packing for HPLC, the liquid crystals are covalently attached to the polysiloxane backbone.

According to Pesek et al. [82–85], the immobilisation of liquid crystals moieties can be performed using the following steps:

1. *silanization* of the surface by a reaction with triethoxysilane to create a hydride intermediate:

- *hydrosilation*, which is carried out in order to attach an organic moiety through formation [82]:
- the ≡Si–C≡ bonds:

cat. = catalyst, typically hexachloroplatinic acid or free radical indicator

(b) the ≡Si–N≡ bonds:  between the silicon containing reactant and the liquid crystalline compound to preserve its liquid crystalline properties. According to Sandoval and Pesek [80], the hydrosilation reaction possesses a great deal of versatility. The catalytic hydrosilation can be employed to attach organic moieties containing a wide variety of functional groups such as alkyl, nitrile, amine, epoxy, etc. to the silicon backbone.
(c) *aminolysis*, i.e., the bonding of low-molecular weight liquid crystal molecules to aminopropyl silica [86]: .

The feasibility of the hydrosilation of a cyanide containing a molecule on a silica hydride surface and the usefulness of such materials as chromatographic stationary phases for HPLC was fully confirmed [82]. Their NMR results indicated the different degrees of association of adjacent bonded moieties and the differences in the retention of PHAs, alkyl-substituted benzenes, and benzo-diazepines.

The hydrosilation of a cyanide containing moietes (i.e., 4-cyano-4′-*n*-pentyl-1,1′ biphenyl and 4-cyano-4′-*n*-pentoxy-1,1′ biphenyl) on a silica hydride surface was possible, and that the bonding of such species for use as chromatographic stationary phases was feasible [82]. The two synthesised column fillings had different chromatographic properties at approximately the same ligand density $( { \approx 0.85 \frac{{\mu {\text{mol}}}}{{{\text{m}}^{2} }}} )$ just as the original liquid crystals had different physical properties, i.e., melting point, clearing point, flow viscosity, etc. [82]. [4-(Allyloxy)benzoyl]-4-methoxyphenol (ABMP) shown also to possess liquid crystal properties when covalently attached to a polysiloxane support.

Generally, the HPLC columns prepared in these ways have very long lifetimes, but the non-specific and specific interactions between adjacent liquid–crystal molecules may be disturbed because of the bonding. In spite of the disturbances and the influence of the mobile phase composition on the structure of the liquid crystal stationary phase, the separations obtained confirm the presence of ordered stationary phases on the siliceous supports, which seem to be the best for attaching liquid-crystalline molecules. The existence of the ordered structure in the bonded state could be proper for the high shape selectivity exhibited in the elution data [86, 87].

The separation properties of the ABMP phase were investigated in detail with the employment of the different mobile-phase compositions, including methanol, acetonitrile, tetrahydrofuran, and water, in various proportions as mobile phases. The polycyclic hydrocarbons (PAHs), carvone, and pulegone were successfully separated at this stationary phase [86].

The values of the logarithmic retention factor (${ \log }k^{'}$) are very often used in study of mobile-phase effects and stationary-phase effects by the solvatochromic comparison method [88]. Pesek and Siouffi obtained the non-linear dependencies for ${ \log }k^{'}$ of chromatographed substances (anthracene, phenanthrene, carvone, and pulegone) versus % organic solvent (tetrahydrofuran, acetonitrile, and methanol) in the mobile phase for ABMP. The ${ \log }k^{'}$ values were much greater than expected at lower percentages of organic in the mobile phase [86]. According to the authors [87], such behaviour was in contrast to the ordinary reverse phase columns which have displayed a linear relationship between the ${ \log }k^{'}$ values and the composition of the mobile phase for each of the compound tested. Nevertheless, each of the compounds used in the tests displayed the linear behaviour under identical conditions when a reverse phase octadecyl column, C-18, was employed [85].

Wise et al. [89] observations of the retention behaviour of PAH isomers on the C-18 column filling were the basis to clarify the nature of the chromatographic separation of the long, narrow molecules of probes on polysiloxanes modified with liquid crystals. Therefore, the property of non-planarity was an extension of the molecular descriptor of the length-to-breadth ratio, and it was the cause that the thickness parameter was taken into account. The retention of linear and non-linear molecules of probes would follow a similar mechanism based on long narrow probes (more linear) having the longest retention. On the basis of the comparison of the elution of analytes obtained previously on the C-18 column [89] and for the ABMP phase [81], the authors suggested that aggregation of the bonded phase began to occur at lower percentages of organic in the mobile phase, and the ABMP material began to assume more ‘liquid crystal-like’ properties. Moreover, the retention of analytes by liquid crystal phases in GC and SFC can be characterised by the so-called ‘slot model’ [87, 89]. According to the ‘slot model’, the relatively planar molecules with the relatively large length/breadth ratios are retained preferentially. Therefore, the shapes of the molecules have a decisive impact on the curvature of the ${ \log }k^{'}$ versus % plot due to the greater force of interaction with the bonded materials creating a ‘slot structure’.

For several years, the MicroSolv Technology Corporation has been offering the columns with chemically bonded liquid crystals as the stationary phases for HPLC [90].

## Conclusions

During the last 10 years, the number of articles connected with the LCSPs testing is lower than in the previous 10 years. The published articles were in majority connected only with gas chromatography. There was no application of liquid crystals as SPs in liquid chromatography; however, the HPLC technique was employed for the testing of results of the synthesis of liquid crystals.

The specific retention volume, $V_{{g(T_{\text{c}} )}}$, is the main magnitude determined in the chromatographic physicochemical tests, which has to be treated as a physicochemical constant. The $V_{{g(T_{\text{c}} )}}$ values depend on the column temperature and the acceptor–donor properties of the system stationary phase–probe molecule.

In the case of the LCSPs, the retention mechanism (in both packed and capillary columns) is closely connected with the solubility of the probe in the stationary phase, its sorption in various mesophases, and its adsorption on the solid LCs. Thus, a statistical description of the sources of the variance associated with the $V_{{g(T_{\text{c}} )}}$ determination is very much needed.

The essential achievements follow on from our review of the literature concerning different liquid crystals (employed as LCSPs) having mesophases with the same structure and revealing both acceptor–donor and separation properties towards the same compositions of analytes can be summed up as follows:

(i) The possibility of the comparison of different LCSP properties towards a relatively large number of analytes (probes) with a large diversity of properties to disclose the most subtle properties of the chromatographic systems tested.
(ii) The employment of the linear solvation energy relationships for the characterisation of LCSPs with different properties.
(iii) The relatively high selectivity properties of poly(propyleneimine)dendrimers connected with the local ordering of the terminal-group shell.
(iv) The relatively high selectivity properties of Schiff’s base LCSPs.
(v) The solution properties of probes in LCSPs can be interpreted employing the Flory liquid crystal model.
(vi) The polysiloxane liquid crystals have similar separation properties as the polycarbosilane-based phases.

In the analytical applications of GC, attention was paid to the fact of how subtle differences in the structures of LCs molecules influenced the ability of the resolution of the mixture of components. It enabled the better recognition of the influence of the LCs and chromatographed substances molecules structures on the resolution effect.

Generally, a satisfactory separation of position isomers with polar substituents and a worse separation of alkyl-substituted compounds affirm the importance of acceptor–donor interactions between the probe used and the LCSPs tested. Moreover, the geometry of the probe molecule (i.e., geometry of molecular orbitals) is also a determinant factor in the retention of each testing substance.

The typical analytical applications of the LCSPs during the last 10 years also observed the IGC tests connected with the mechanisms of interactions of the LCSP—eluting substance. The values of the thermodynamic magnitudes, such as enthalpies and entropies of solution, activity coefficients of chromatographed substances and interaction parameters were determined by the IGC method. It enabled a better understanding of the interdependencies of the LCs structures and their properties while used as LCSPs.

The comparison of the results of the IGC and other techniques leads to the conclusion that the IGC is a very useful technique of the LCs properties testing, namely the temperatures of phase transitions.

At the transition temperatures, the differences of the Flory–Huggins interaction parameter can be related to the ‘disorder parameter’. This apart, the differences in the ability of one another and each other interaction between the molecules of testing substances and LCs molecules are regarded to be the source of the deviation from the constant values of the activity coefficients. The selectivity of the LCSPs connected with the solution properties of the chromatographed substances toward these phases can be interpreted employing the Flory liquid crystal model, and the differences in the values of the interaction parameter can be attributed to the variation in the testing substance access to functional groups within the mesophase structures.

The absorption and selective properties of the liquid-crystalline and isotropic phases of the dendrimers were significantly circumscribed by the steric factors, in all probability related to the efficacy of penetration of the free volume between the conformational disordered alkyl chains of dendrimer macromolecules by the molecules of the testing substances. In this context, it is necessary to emphasise that the terminal groups of the dendrimers considerably affect their utile chromatographic properties.
